# The Thyroid Hormone Transporter Mct8 Restricts Cathepsin-Mediated Thyroglobulin Processing in Male Mice through Thyroid Auto-Regulatory Mechanisms That Encompass Autophagy

**DOI:** 10.3390/ijms22010462

**Published:** 2021-01-05

**Authors:** Vaishnavi Venugopalan, Alaa Al-Hashimi, Maren Rehders, Janine Golchert, Vivien Reinecke, Georg Homuth, Uwe Völker, Mythili Manirajah, Adam Touzani, Jonas Weber, Matthew S. Bogyo, Francois Verrey, Eva K. Wirth, Ulrich Schweizer, Heike Heuer, Janine Kirstein, Klaudia Brix

**Affiliations:** 1 Department of Life Sciences and Chemistry, Focus Area HEALTH, Jacobs University Bremen, Campus Ring 1, D-29759 Bremen, Germany; v.venugopalan@jacobs-university.de (V.V.); a.alhashimi@jacobs-university.de (A.A.-H.); m.rehders@jacobs-university.de (M.R.); mythilimanirajah@gmail.com (M.M.); adamtouzani1996@gmail.com (A.T.); jweber@cisbio.com (J.W.); 2 Department of Functional Genomics, Interfaculty Institute for Genetics and Functional Genomics, University Medicine Greifswald, Felix-Hausdorff-Str. 8, 17475 Greifswald, Germany; janine.golchert@med.uni-greifswald.de (J.G.); vivienreinecke@web.de (V.R.); georg.homuth@uni-greifswald.de (G.H.); voelker@uni-greifswald.de (U.V.); 3 Department of Pathology, School of Medicine, Stanford University, 300 Pasteur Dr., Stanford, CA 94305-5324, USA; mbogyo@stanford.edu; 4 Physiologisches Institut, Universität Zürich, Winterthurerstr. 190, CH-8057 Zürich, Switzerland; francois.verrey@uzh.ch; 5 Berlin Institute of Health, Department of Endocrinology and Metabolism, Charité—Universitätsmedizin Berlin, Corporate Member of Freie Universität Berlin, Humboldt-Universität zu Berlin, Hessische Str. 3-4, Germany and DZHK (German Centre for Cardiovascular Research), Partner Site Berlin, D-10115 Berlin, Germany; eva.wirth@charite.de; 6 Institut für Biochemie und Molekularbiologie, Medizinische Fakultät, Universität Bonn, Nußallee 11, D-53115 Bonn, Germany; uschweiz@uni-bonn.de; 7 Klinik für Endokrinologie, Diabetologie und Stoffwechsel, Universitätsklinikum Essen (AöR), Universität Duisburg-Essen, Hufelandstr. 55, D-45147 Essen, Germany; heike.heuer@uk-essen.de; 8 Fachbereich 2 Biologie/Chemie, Faculty of Cell Biology, Universität Bremen, Leobener Straße 5, D-28359 Bremen, Germany; kirstein@uni-bremen.de

**Keywords:** autophagy, cathepsins, lysosomal biogenesis, monocarboxylate transporter 8, thyroid auto-regulation

## Abstract

The thyroid gland is both a thyroid hormone (TH) generating as well as a TH responsive organ. It is hence crucial that cathepsin-mediated proteolytic cleavage of the precursor thyroglobulin is regulated and integrated with the subsequent export of TH into the blood circulation, which is enabled by TH transporters such as monocarboxylate transporters Mct8 and Mct10. Previously, we showed that cathepsin K-deficient mice exhibit the phenomenon of functional compensation through cathepsin L upregulation, which is independent of the canonical hypothalamus-pituitary-thyroid axis, thus, due to auto-regulation. Since these animals also feature enhanced Mct8 expression, we aimed to understand if TH transporters are part of the thyroid auto-regulatory mechanisms. Therefore, we analyzed phenotypic differences in thyroid function arising from combined cathepsin K and TH transporter deficiencies, i.e., in *Ctsk*^-/-^/*Mct10*^-/-^, *Ctsk*^-/-^/*Mct8*^-/y^, and *Ctsk*^-/-^/*Mct8*^-/y^/*Mct10*^-/-^. Despite the impaired TH export, thyroglobulin degradation was enhanced in the mice lacking Mct8, particularly in the triple-deficient genotype, due to increased cathepsin amounts and enhanced cysteine peptidase activities, leading to ongoing thyroglobulin proteolysis for TH liberation, eventually causing self-thyrotoxic thyroid states. The increased cathepsin amounts were a consequence of autophagy-mediated lysosomal biogenesis that is possibly triggered due to the stress accompanying intrathyroidal TH accumulation, in particular in the *Ctsk*^-/-^/*Mct8*^-/y^/*Mct10*^-/-^ animals. Collectively, our data points to the notion that the absence of cathepsin K and Mct8 leads to excessive thyroglobulin degradation and TH liberation in a non-classical pathway of thyroid auto-regulation.

## 1. Introduction

The tasks of the thyroid gland are enabled by its functional units, the thyroid follicles, comprising of thyroid epithelial cells—thyrocytes—arranged in a sphere-forming monolayer surrounding the thyroid follicle lumen, wherein thyroglobulin (Tg), the precursor of thyroid hormones (TH), is compacted and stored [1,2,3]. The thyroid gland is a highly vascularized organ, in which thyroid follicles and the blood vessels surrounding them are collectively known to function as the angio-follicular units (AFU) [4]. The thyroid gland is canonically regulated by the hypothalamus-pituitary-thyroid (HPT) axis, in that a demand of TH induces the hypothalamus to release the thyrotropin-releasing hormone (TRH) that subsequently triggers the anterior pituitary gland to release thyroid stimulating hormone (TSH), eventually resulting in the activation of thyrocytes by binding to their basolateral TSH receptors, in order to restore TH levels in the bloodstream and target tissues [5,6]. In response to TSH stimulation, specific proteolytic enzymes called cysteine cathepsins are transported from endo-lysosomes of thyrocytes to the apical plasma membrane and are secreted into the extracellular space, where they exert their proteolytic functions for the solubilization of luminal Tg [3,7,8]. Partially solubilized Tg molecules are re-internalized by the thyrocytes, where they are further degraded, within endo-lysosomal compartments, to liberate TH, i.e., 3,3′,5,5′ tetraiodo-L-thyronine (T4) and the biologically active form 3′,3,5 triiodothyronine (T3) [9]. TH are released into the blood circulation by means of thyroid hormone transmembrane transporters such as monocarboxylate transporters Mct8 and Mct10/TAT1, which also mediate the uptake of TH in peripheral organs [10,11,12,13,14]. Tissues dependent on TH include the cardiovascular system, the central nervous system, the intestine, the liver, the skeleton, and the thyroid gland itself, thereby specifying the thyroid gland as a TH releasing as well as TH responding organ [9]. Thyroid dysfunction arising from disturbed TH generation, TH release, or TH action poses as the most common form of endocrine diseases [15,16]. It should be noted, however, that while low TH concentrations in target cells can interrupt its normal function, increased accumulation of TH within tissues also leads to profound adverse effects [17]. Hence, it is important to restrict uncontrolled Tg proteolysis by cathepsins to not only maintain appropriate serum TH levels but also intracellular TH levels in all target tissues, including the thyroid gland [18].

Previous studies have shown that a deficiency of one cathepsin can be compensated with an increased expression of another cathepsin in a mechanism of functional redundancy [19]. A specific example of this phenomenon is the upregulation of cathepsin L in the thyroid gland of cathepsin K-deficient (*Ctsk*^-/-^) mice, consequently maintaining regular serum TH levels [20]. Since TH liberation is classically regulated by the HPT axis, it was thought to be plausible that the upregulation of cathepsin L, and thereby the exhibited functional redundancy for the loss of cathepsin K, is also under the control of the HPT axis. However, we have shown recently that in these mice the serum TSH levels remain normal as in wild type (WT) control mice [21]. This indicates that the altered Tg-processing abilities (via cathepsin L upregulation) in *Ctsk*^-/-^ mice is not a consequence of altered serum thyroid status. Furthermore, we have shown that in this murine model, maintenance of normal serum TH levels in *Ctsk*^-/-^ mice could be attributed to the enhanced expression of the Mct8 transporter at the basolateral plasma membrane of *Ctsk*^-/-^ thyrocytes, allowing for increased export of T4 from the thyroid gland into the circulation [18]. Therefore, we postulate the existence of intrathyroidal TH-sensing mechanisms that regulate and integrate TH liberation and release from thyroid follicles to not only meet the TH necessities of peripheral organs but also those of the thyroid gland itself, and that such auto-regulatory mechanisms may be independent of the HPT axis. 

In the current study, we aim to understand whether Mct8 and/or its structurally closest relative Mct10 is involved in the non-canonical regulation of cathepsin-mediated Tg proteolysis. Therefore, we have created murine models lacking Mct8 and/or Mct10 in addition to cathepsin K, i.e., *Ctsk*^-/-^/*Mct10*^-/-^, *Ctsk*^-/-^/*Mct8*^-/y^, and *Ctsk*^-/-^/*Mct8*^-/y^/*Mct10*^-/-^. Using these models, we evaluate the parameters that contribute to the functionality of the thyroid gland, namely, Tg cross-linking and degradation, expression levels of Tg-processing proteases, i.e., cathepsins, and their proteolytic activities. We have restricted our analyses to male mice, only, because it is well-known that gender- or sex-specific differences in thyroid homeostasis occur [22,23,24]. Fluctuations of TH concentrations and regulated body functions with the estrous cycle are prevalent in females, and can only be properly studied in ovariectomized and hormone-substituted animals [24,25]. However, the effects of such manipulations would likely interfere differently in distinct murine genotypes making conclusions on either or both, genotypic or sex-dependent changes, difficult. Moreover, global knock-out models were preferred in this study over thyroid-specific animal models due to availability and to not risk any issues with limited penetrance [13,26].

Our data indicate that while the thyroid phenotype is largely unaltered in *Ctsk*^-/-^ animals, mice lacking Mct8 in a cathepsin K-deficient background, particularly the triple-deficient genotype, i.e., *Ctsk*^-/-^/*Mct8*^-/y^/*Mct10*^-/-^, are differentially and significantly affected in thyroid gland functionality. Although TH export is impaired in these mice, their thyrocytes continue to degrade Tg as a result of increased protein amounts and proteolytic activities of cathepsins, which in turn is the consequence of induced lysosomal biogenesis mediated by autophagy. Eventually, the excessive Tg degradation, combined with the defect in exporting TH from thyroid follicles, leads to intrathyroidal TH accumulation and thyrotoxicity that is possibly responsible in the furtherance of the stress-induced autophagy and persistent Tg proteolysis.

## 2. Results

### 2.1. Tg Storage Status Is Altered in Combined Cathepsin K and TH Transporter Deficiency

**Figure 1:**
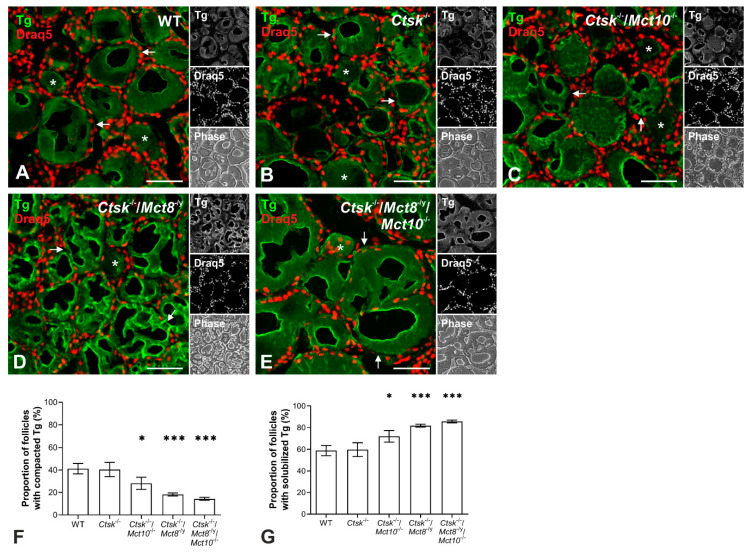
Thyroglobulin storage status in combined cathepsin K and TH transporter deficiency. Thyroid cryo-sections were immunolabelled with antibodies against Tg (green), and the intraluminal Tg status was assessed in (**A**) WT, (**B**) *Ctsk*^-/-^, (**C**) *Ctsk*^-/-^/*Mct10*^-/-^, (**D**) *Ctsk*^-/-^/*Mct8*^-/y^, and (**E**) *Ctsk*^-/-^/*Mct8*^-/y^/*Mct10*^-/-^ mice by confocal laser scanning microscopy. Single-channel fluorescence and corresponding phase contrast micrographs are depicted in the right panels as indicated. Tg staining in the lumen was either homogenous and rather faint, corresponding to tightly compacted Tg (asterisks), or appeared multilayered, referring to solubilized Tg (arrows), depending on the accessibility of intraluminal Tg for binding of the Tg-specific antibodies. Bar graphs indicate the proportion of follicles displaying compacted (**F**) and solubilized Tg (**G**) relative to the total number of investigated follicles, respectively, in the genotypes. Mice lacking both, cathepsin K and TH transporters, showed a decrease in the number of follicles with compacted Tg, and accordingly an increase in the number of follicles exhibiting Tg in multilayers. Animals analyzed: *n* = 3 per genotype, numbers of follicles analyzed: *n* = 1151, 688, 633, 743, and 852 for WT, *Ctsk*^-/-^, *Ctsk*^-/-^/*Mct10*^-/-^, *Ctsk*^-/-^/*Mct8*^-/y^, and *Ctsk*^-/-^/*Mct8*^-/y^/*Mct10*^-/-^ mice, respectively. Nuclei were counter-stained with Draq5^TM^ (red). Scale bars represent 50 µm. Data is depicted as means ± SD. Levels of significance are indicated as * for *p* < 0.05 and *** for *p* < 0.001.

Following biosynthesis and post-translational modification, Tg becomes iodinated, covalently cross-linked, and Tg multimers are deposited in the thyroid follicle lumen as so-called ‘thyroid globules’ at high protein concentrations of up to 800 mg/mL [3,27,28,29]. The extent of Tg compaction in the thyroid follicle lumina is heterogeneous, thereby giving rise to a typical multilayered appearance [18]. To determine the Tg storage appearance in the follicle lumen, tissue sections from the thyroid glands of WT, *Ctsk*^-/-^, *Ctsk*^-/-^/*Mct10*^-/-^, *Ctsk*^-/-^/*Mct8*^-/y^, and *Ctsk*^-/-^/*Mct8*^-/y^/*Mct10*^-/-^ mice were analyzed by immunofluorescence using Tg-specific antibodies. Partially solubilized or degraded Tg produces higher signal intensities, because processed Tg is more easily accessible for binding of the Tg-specific antibodies, whereas a rather homogenous signal over the follicle lumen is seen when it contains compacted and cross-linked Tg [3,18,20]. Based on this notion, micrographs of immuno-stained thyroid sections were manually assessed to enumerate the thyroid follicles with tightly compacted (Figure 1A–E, asterisks) or multilayered Tg (arrows). Results revealed that in *Ctsk*^-/-^ mice, 40.4% ± 6.3 of the follicles contained compacted Tg, while 59.6% ± 6.3 of the follicles contained Tg in multilayers (Figure 1B,F,G). Thus, the proportions of thyroid follicles with cross-linked and solubilized Tg in *Ctsk*^-/-^ were similar to those found in WT controls (41.2% ± 4.6 and 58.8% ± 4.6, respectively) (Figure 1A,F,G). Upon additional loss of Mct10, an increase in the proportion of follicles with multilayered Tg appearance was observed in *Ctsk*^-/-^/*Mct10*^-/-^, while fewer follicles exhibited homogenous Tg staining, representing compacted Tg (71.9% ± 5.4 and 28.1% ± 5.4, respectively) (Figure 1C,F,G). The effect of fewer follicles with cross-linked Tg became more pronounced, when Mct8 or both Mct8 and Mct10 were lacking, i.e., 81.8% ± 1.3 and 85.6% ± 1.5 of follicles exhibited luminal Tg in multilayers and concentric circles, while 18.2% ± 1.3 and 14.4% ± 1.5 of the follicles revealed uniform Tg staining, respectively, in thyroid tissue of *Ctsk*^-/-^/*Mct8*^-/y^ and *Ctsk*^-/-^/*Mct8*^-/y^/*Mct10*^-/-^ mice (Figure 1D–G). The enhanced accessibility of intraluminal Tg to the anti-Tg antibodies, as evidenced by intense and predominantly multilayered immunofluorescence signals in *Ctsk*^-/-^/*Mct8*^-/y^ and *Ctsk*^-/-^/*Mct8*^-/y^/*Mct10*^-/-^ thyroid follicles suggested that Tg solubilization was altered in these animals.

**Figure 2:**
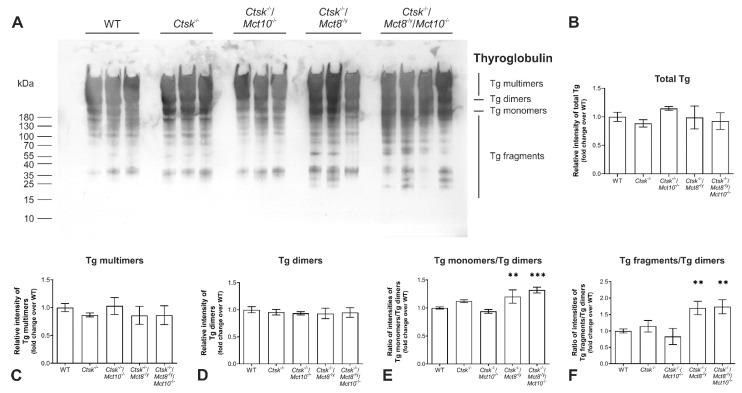
Thyroglobulin cross-linkage in combined cathepsin K and TH transporter deficiencies. Proteins isolated from whole thyroid tissue lysates of the indicated genotypes and WT controls were separated on 8–18% horizontal SDS-gels under non-reducing conditions, transferred onto nitrocellulose membrane, and immunoblotted using Tg-specific antibodies. A representative immunoblot is shown (**A**). The molecular mass markers are given in the left margin. Bands representing multimers, dimers, monomers, and fragments of Tg are indicated in the right margin. Bar charts (**B**–**E**) represent densitometry analyses of total Tg (**B**), Tg multimers (**C**), Tg dimers (**D**), Tg monomers/Tg dimers (**E**), and Tg fragments/Tg dimers (**F**) in the investigated genotypes as fold changes over WT controls. No genotypic differences in band intensities of Tg multimers or Tg dimers were observed, indicating that Tg cross-linkage most likely remained unaltered upon cathepsin K and/or TH transporter deficiencies. The ratio of band intensities of Tg monomers over Tg dimers (representing Tg solubilization), as well as Tg fragments over Tg dimers (representing Tg degradation) showed an increase in *Ctsk*^-/-^/*Mct8*^-/y^ and *Ctsk*^-/-^/*Mct8*^-/y^/*Mct10*^-/-^ thyroid tissue, suggesting enhanced solubilization and altered proteolytic processing of Tg. Animals analyzed: *n* = 3–4 per genotype. Densitometry data was normalized to total Ponceau-stained protein per lane and is depicted as means ± SD. Levels of significance are indicated as ** for *p* < 0.01 and *** for *p* < 0.001.

To study the genotypic effect on the Tg status in further detail, we analyzed Tg cross-linking and degradation states by immunoblotting. Therefore, whole tissue lysates from the thyroid glands of WT, *Ctsk*^-/-^, *Ctsk*^-/-^/*Mct10*^-/-^, *Ctsk*^-/-^/*Mct8*^-/y^, and *Ctsk*^-/-^/*Mct8*^-/y^/*Mct10*^-/-^ mice were separated by SDS-PAGE under non-reducing conditions to preserve disulfide bonds between the molecules, thereby keeping Tg multimers intact. Separated proteins were transferred onto nitrocellulose membrane, and immunoblotted with antibodies against Tg. All Tg forms, i.e., Tg multimers, dimers, or monomers were observed along with Tg degradation fragments in all investigated genotypes to different extents (Figure 2A). Densitometry analysis was performed by normalizing the density of the immuno-stained bands to the amount of total Ponceau-stained proteins per lane. Results revealed that amounts of total Tg were similar between the investigated genotypes and no significant differences were observed (Figure 2B). The density of bands corresponding to Tg multimers was comparable with WT controls and between all analyzed genotypes (Figure 2C). Similarly, no differences were observed in the relative band intensities of Tg dimers (Figure 2D). Hence, these data suggested that Tg cross-linking was unaltered in mice lacking cathepsin K as well as in combined cathepsin K and TH transporter deficiencies. However, although the amounts of Tg dimers appeared to be similar in all genotypes analyzed, the ratio of monomeric to dimeric Tg showed a significant increase in *Ctsk*^-/-^/*Mct8*^-/y^ and *Ctsk*^-/-^/*Mct8*^-/y^/*Mct10*^-/-^ mice in comparison to WT controls (Figure 2E). Likewise, the ratio of band densities of Tg fragments to Tg dimers was enhanced in these genotypes (Figure 2F).

The results indicated enhanced solubilization of monomeric Tg and increased proteolytic Tg processing upon Mct8 single and Mct8/Mct10 double deficiencies, while the total amounts of Tg and its multimerization remained unaltered. We propose that Tg turnover in the analyzed mouse models might be affected by disbalances in proteases that play a role in Tg solubilization and utilization rather than by affecting Tg cross-linking.

### 2.2. Tg Degradation Remains Normal upon Cathepsin K Deficiency, but Is Enhanced in Ctsk^-/-^/Mct8^-/y^ and Ctsk^-/-^/Mct8^-/y^/Mct10^-/-^ Mice

**Figure 3:**
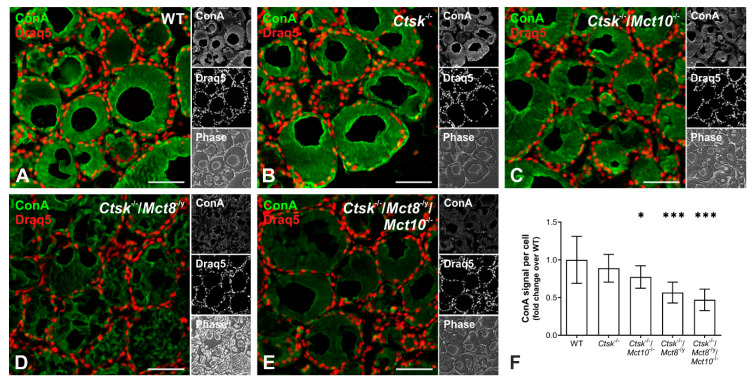
Protein glycosylation in combined cathepsin K and TH transporter deficiencies. Thyroid tissue sections from (**A**) WT, (**B**) *Ctsk*^-/-^, (**C**) *Ctsk*^-/-^/*Mct10*^-/-^, (**D**) *Ctsk*^-/-^/*Mct8*^-/y^, and (**E**) *Ctsk*^-/-^/*Mct8*^-/y^/*Mct10*^-/-^ mice were stained with biotinylated lectin ConA and Alexa 546-conjugated streptavidin (green) to assess any difference in glycosylation states by confocal laser scanning microscopy. Merged, single-channel fluorescence, and corresponding phase contrast micrographs are displayed as indicated. The intensity of ConA staining was measured using a Cell Profiler pipeline and normalized to the numbers of cells (**F**). Mice lacking both cathepsin K and either or both TH transporters showed a significant decrease in the ConA signal. Animals analyzed: *n* = 3 per genotype with 8–10 micrographs quantified per animal, respectively. Nuclei were counter-stained with Draq5^TM^ (red). Scale bars represent 50 µm. Data is depicted as fold changes over WT controls as means ± SD. Levels of significance are indicated as * for *p* < 0.05 and *** for *p* < 0.001.

Tg undergoes both N- and O-linked glycosylation, wherein up to 10% of its molecular mass is contributed by complex high-mannose and glucosamine carbohydrate moieties [30,31]. Because the extent of glycosylation is indicative of the structural integrity of glycosylated molecules, we thought to investigate the glycosylation states by staining thyroid tissue sections with ConA, a lectin that specifically binds to α-D-mannosyl α-D-glucosyl groups [32] and typically detects both cellular and luminal glycoconjugates in murine thyroid tissue [33]. The ConA-positive fluorescence signals were quantified using a Cell Profiler-based pipeline to evaluate the extent of glycosylation in the different genotypes (Figure 3A–F). In comparison to WT thyroid tissue, intensity measurements of ConA signals were unaltered in *Ctsk*^-/-^ mice, however, they were diminished in *Ctsk*^-/-^/*Mct10*^-/-^ and further reduced in *Ctsk*^-/-^/*Mct8*^-/y^ and *Ctsk*^-/-^/*Mct8*^-/y^/*Mct10*^-/-^ thyroid cryo-sections (Figure 3F).

These results indicated that deficiencies in both cathepsin K and TH transporters, i.e., Mct8 and/or Mct10, affect protein glycosylation in the thyroid gland. It cannot be excluded at this point that glycosylation of other thyrocyte proteins are altered as well. However, ConA staining is most prominent over the follicular lumen, consistent with the notion of Tg being the main secretory product of thyrocytes, and is hence considered a proxy for Tg glycosylation states. The decrease in ConA-positive signals observed upon combined cathepsin K and TH transporter deficiencies suggested that Tg may be less stable and hence more prone to degradation in these genotypes when compared to WT or *Ctsk*^-/-^ mice.

**Figure 4:**
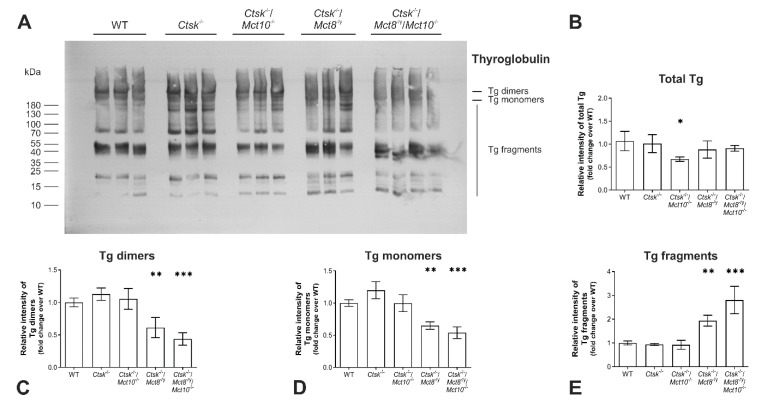
Gross thyroglobulin degradation states in thyroid glands of mice lacking cathepsin K and TH transporters. Whole thyroid tissue lysates of indicated genotypes and WT controls were separated on 8–18% horizontal SDS-gels under reducing conditions, transferred onto nitrocellulose membrane, and subsequently probed with anti-Tg antibodies. Shown is a representative immunoblot (**A**). The molecular mass markers are given in the left margin. Bands representing dimers, monomers, and fragments of Tg are indicated in the right margin of the immunoblot. Bar charts (**B**–**E**) represent densitometry analyses of total Tg (**B**), Tg dimers (**C**), Tg monomers (**D**), and Tg fragments (**E**) in the investigated genotypes as fold changes over WT controls. *Ctsk^-^*^/-^/*Mct8*^-/y^ and *Ctsk*^-/-^/*Mct8*^-/y^/*Mct10*^-/-^ mice showed a decrease in mono- and dimeric Tg amounts, while exhibiting an increase in the amounts of Tg fragments, thereby indicating enhanced Tg degradation. Animals analyzed: *n* = 3–4 per genotype. Densitometry data was normalized to total Ponceau-stained protein per lane and is depicted as means ± SD. Levels of significance are indicated as * for *p* < 0.05, ** for *p* < 0.01, and *** for *p* < 0.001.

Therefore, next we sought out to compare the Tg degradation status in *Ctsk*^-/-^, *Ctsk*^-/-^/*Mct10*^-/-^, *Ctsk*^-/-^/*Mct8*^-/y^, and *Ctsk*^-/-^/*Mct8*^-/y^/*Mct10*^-/-^ mice to their WT littermates by SDS-PAGE under reducing conditions and immunoblotting using Tg-specific antibodies (Figure 4A). Results showed that the total Tg protein amounts were comparable between all genotypes, except in thyroid lysates of *Ctsk*^-/-^/*Mct10*^-/-^ mice, wherein a decrease of approx. 30% in total Tg was observed relative to WT controls (Figure 4B). It remains unclear why less total Tg was observed in the *Ctsk*^-/-^/*Mct10*^-/-^ animals. However, the abundance of Tg dimers, monomers, and fragments in *Ctsk*^-/-^ and *Ctsk*^-/-^/*Mct10*^-/-^ thyroid lysates was similar to that of the WT animals. Interestingly, densitometry analysis of the bands representing soluble Tg dimers and monomers revealed a decline in *Ctsk*^-/-^/*Mct8*^-/y^, which became more prominent in *Ctsk*^-/-^/*Mct8*^-/y^/*Mct10*^-/-^ thyroid lysates. (Figure 4C,D, respectively). Consequently, an increase in the amounts of soluble Tg fragments was observed in *Ctsk*^-/-^/*Mct8*^-/y^ and, even more so, in *Ctsk*^-/-^/*Mct8*^-/y^/*Mct10*^-/-^ thyroid lysates, while the overall Tg degradation states in *Ctsk*^-/-^ and *Ctsk*^-/-^/*Mct10*^-/-^ were comparable to that in WT controls (Figure 4E). 

The decrease in the intensities of dimeric and monomeric Tg and the corresponding increase in Tg fragments further support the notion that Tg solubilization and subsequent proteolytic utilization for TH liberation is enhanced when either Mct8 or both Mct8 and Mct10 are lacking in a cathepsin K-deficient background. Hence, the results further supported the notion that Tg solubilization and degradation are affected in particular in combined cathepsin K and Mct8 deficiencies (compare with Figure 2).

### 2.3. Proteolytic Activities of Cysteine Peptidases Are Enhanced in Ctsk^-/-^/Mct8^-/y^ and Ctsk^-/-^/Mct8^-/y^/Mct10^-/-^ Mice

**Figure 5:**
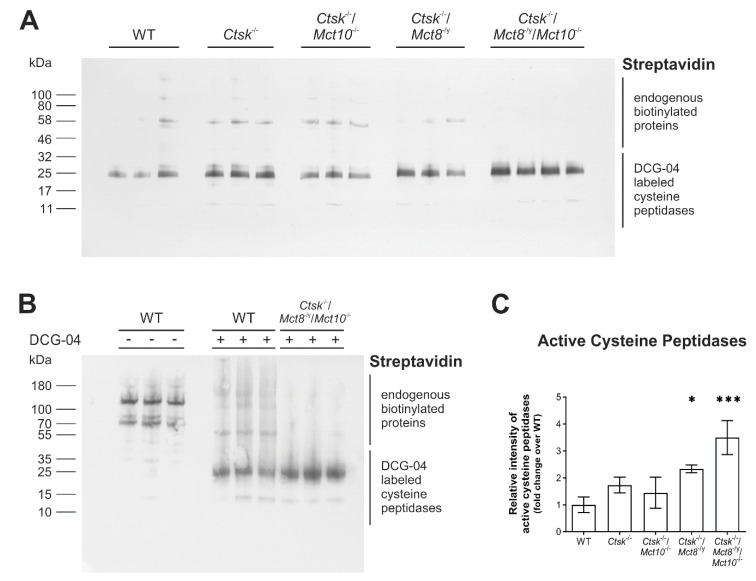
Active cysteine peptidases in thyroid glands of mice lacking cathepsin K alone or in combination with TH transporter deficiencies. Whole thyroid tissue from WT, *Ctsk*^-/-^, *Ctsk*^-/-^/*Mct10*^-/-^, *Ctsk*^-/-^/*Mct8*^-/y^ and *Ctsk*^-/-^/*Mct8*^-/y^/*Mct10*^-/-^ mice was homogenized in lysis buffer containing 5 µM biotinylated activity-based probe DCG-04 before protein separation and staining of the blots with HRP-conjugated streptavidin to exclusively detect proteolytically active cysteine peptidases (**A**). Molecular mass markers are indicated in the left margin. Controls were conducted with WT tissue that was lysed without the addition of DCG-04, demonstrating streptavidin detection of some high molecular mass bands representing endogenous biotinylated proteins (**B**). However, these are most likely not cysteine peptidases ranging from 20–30 kDa in molecular mass. Therefore, the densities of the resulting bands below the 32-kDa molecular mass marker were normalized to total Ponceau-stained protein per lane, and are presented as fold changes over WT (**C**). It is important to note that the DCG-04 activity-based probe binds in equimolar ratio to active cysteine peptidases, only. The quantitation of signal intensities is therefore representative of proteolytic activity. The relative signal intensity of active cysteine peptidases was higher in thyroid lysates of *Ctsk*^-/-^/*Mct8*^-/y^ and further enhanced in *Ctsk*^-/-^/*Mct8*^-/y^/*Mct10*^-/-^ mice in comparison to WT controls (**C**). Animals analyzed: *n* = 3–4 per genotype. Data is depicted as means ± SD. Levels of significance are indicated as * for *p* < 0.05 and *** for *p* < 0.001.

TH synthesized on the Tg backbone are liberated through proteolytic cleavage by peptidases [7,18], many of which belong to the family of cysteine cathepsins, of which 18 different ones are expressed in mice [34]. We hence wanted to determine whether increased Tg degradation in the TH transporter-deficient murine models could be explained by altered activities of Tg-processing enzymes. Therefore, thyroid tissue lysates were prepared in lysis buffer containing biotinylated DCG-04, an activity-based probe that specifically binds in equimolar fashion to proteolytically active cysteine peptidases. Lysates prepared without the inclusion of biotinylated-DCG-04 served as a control for endogenous biotinylated proteins. Proteins were separated on horizontal gradient SDS-gels, transferred to nitrocellulose membrane and incubated with HRP-conjugated streptavidin to visualize DCG-04 reacted proteins. Results showed that bands depicting endogenous biotinylated proteins run above those representing mature and proteolytically active cysteine cathepsins (Figure 5A,B). Densitometry analysis of the blots in comparison with WT controls showed a trend toward higher signal intensity of active cysteine proteases in *Ctsk*^-/-^ mice, although this did not reach statistical significance (Figure 5C). However, an additional lack of Mct8 led to a significant rise in the amounts of DCG-04 reacted cysteine peptidases in *Ctsk*^-/-^/*Mct8*^-/y^, displaying a 2.3-fold increase over WT controls (Figure 5C). Enhancement in levels of active cysteine proteases was most pronounced in the triple knockout genotype *Ctsk*^-/-^/*Mct8*^-/y^/*Mct10*^-/-^ (3.5-fold increase) (Figure 5C), and can be correlated to the extents of Tg solubilization and degradation, which were most accentuated in these animals (cf. Figure 4). The data suggested that the altered Tg degradation states detected in combined cathepsin K and TH transporter deficiencies are a result of increased proteolytic activities of cysteine peptidases in the thyroid gland of the respective mice. However, it cannot be concluded on the activity of a specific cysteine peptidase by using this approach.

### 2.4. Increased Proteolytic Activity of Cysteine Peptidases Is Not Due to an Imbalance of Proteolytic and Anti-Proteolytic Factors

**Figure 6:**
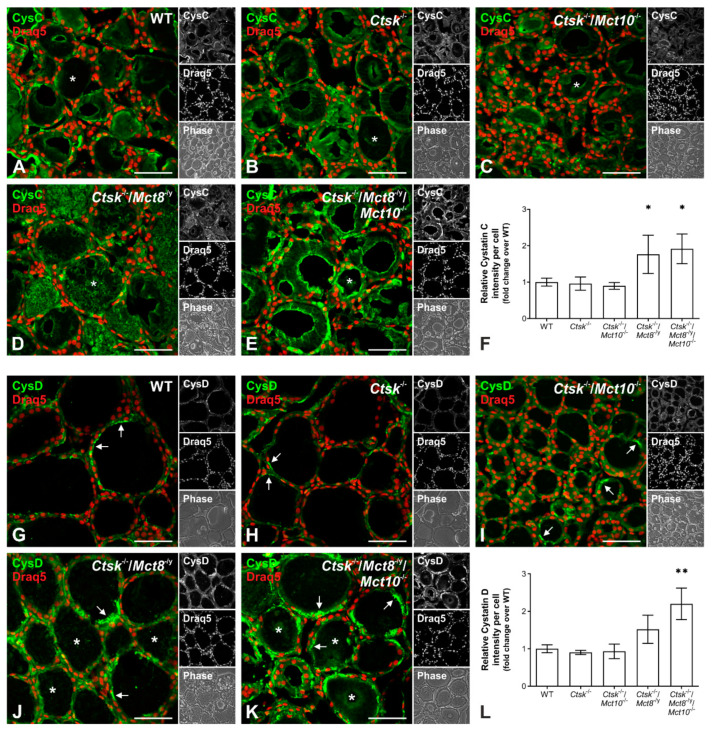
Cystatin C and D levels in combined cathepsin K and TH transporter deficiencies. Thyroid cryo-sections from mice of the indicated genotypes and WT controls were stained with antibodies specific for cystatin C (**A**–**E**) or cystatin D (**G**–**K**) (green). Merged, single-channel fluorescence, and corresponding phase contrast micrographs are displayed as indicated. Micrographs show that cystatin C predominantly localized to the thyroid follicle lumen (**A**–**E**, asterisks) while cystatin D mainly localized to the apical pericellular space of thyrocytes (**G**–**K**, arrows) in all genotypes investigated. Intraluminal cystatin C (**D** and **E**, asterisks) and D (**J** and **K**, asterisks) signals appeared enhanced in mice lacking Mct8. The intensities of cystatin C and D staining were measured using a Cell Profiler pipeline and normalized to the numbers of cells (**F** and **L**, respectively). *Ctsk*^-/-^/*Mct8*^-/y^/*Mct10*^-/-^ mice showed a two-fold increase in thyroidal cystatin C and D signals when compared to WT controls. Animals analyzed: *n* = 3 per genotype with 7–12 micrographs quantified per animal, respectively. Nuclei were counter-stained with Draq5^TM^ (red). Scale bars represent 50 µm. Data is depicted as fold changes over WT controls as means ± SD. Levels of significance are indicated as * for *p* < 0.05 and ** for *p* < 0.01.

The activity of cysteine proteases is counter-balanced by endogenous inhibitors such as cystatins. Hence, it is possible that a reduction in their expression could reflect an enhanced cysteine protease activity. To test this hypothesis, cryo-sections of thyroids from WT, *Ctsk*^-/-^, *Ctsk*^-/-^/*Mct10*^-/-^, *Ctsk*^-/-^/*Mct8*^-/y^, and *Ctsk*^-/-^/*Mct8*^-/y^/*Mct10*^-/-^ mice were labeled with antibodies against cystatin C or D, and micrographs were analyzed for immunostaining intensities using Cell Profiler. In all analyzed genotypes, cystatins C (Figure 6A–E) and D (Figure 6G–K) were mainly localized to the thyroid follicle lumen (asterisks), while cystatin D signals were especially prominent in the apical pericellular space of thyrocytes (Figure 6G–K, arrows). Quantification of immuno-positive signals indicated that cystatin C and D levels in *Ctsk*^-/-^ and *Ctsk*^-/-^/*Mct10*^-/-^ thyroid glands were comparable to WT controls (Figure 6F,L). In contrast, *Ctsk*^-/-^/*Mct8*^-/y^ and *Ctsk*^-/-^/*Mct8*^-/y^/*Mct10*^-/-^ showed significant two-fold increases over WT controls in thyroidal cystatin C signals (Figure 6F). In addition, staining intensity measurements showed that cystatin D was enhanced in the genotypes that lack Mct8, although this effect became statistically significant only when both Mct8 and Mct10 were missing (Figure 6L). The results indicated that enhanced proteolytic activities were not counter-balanced by decreased inhibitor amounts, and vice versa, thus pointing to an overall upregulation of the Tg-degrading enzymes and their endogenous inhibitors in TH transporter-deficient mouse thyroid glands.

Since *Ctsk*^-/-^ and *Ctsk*^-/-^/*Mct10*^-/-^ mice showed only mild or insignificant differences in the amounts of active cysteine proteases (cf. Figure 5) and no changes in cystatin levels (Figure 6F,L), the results confirmed the interpretation that the overall balance of proteolytic and anti-proteolytic activities is obviously not affected to an extent that Tg degradation would become altered in these genotypes. Surprisingly and despite the enhancement of cystatins C and D signals in the thyroid follicles of *Ctsk*^-/-^/*Mct8*^-/y^ and *Ctsk*^-/-^/*Mct8*^-/y^/*Mct10*^-/-^ mice (Figure 6F,L), increased activities of cysteine proteases (cf. Figure 5) and corresponding enhanced Tg degradation (cf. Figure 4) was observed, thereby suggesting cathepsin maturation states might be at misbalance when TH transporters are lacking in a cathepsin K-deficient background.

### 2.5. Processing States of Tg-Processing Cathepsins Remain Unaltered in Thyroid Tissue of Ctsk^-/-^/Mct8^-/y^ and Ctsk^-/-^/Mct8^-/y^/Mct10^-/-^ Mice

**Figure 7:**
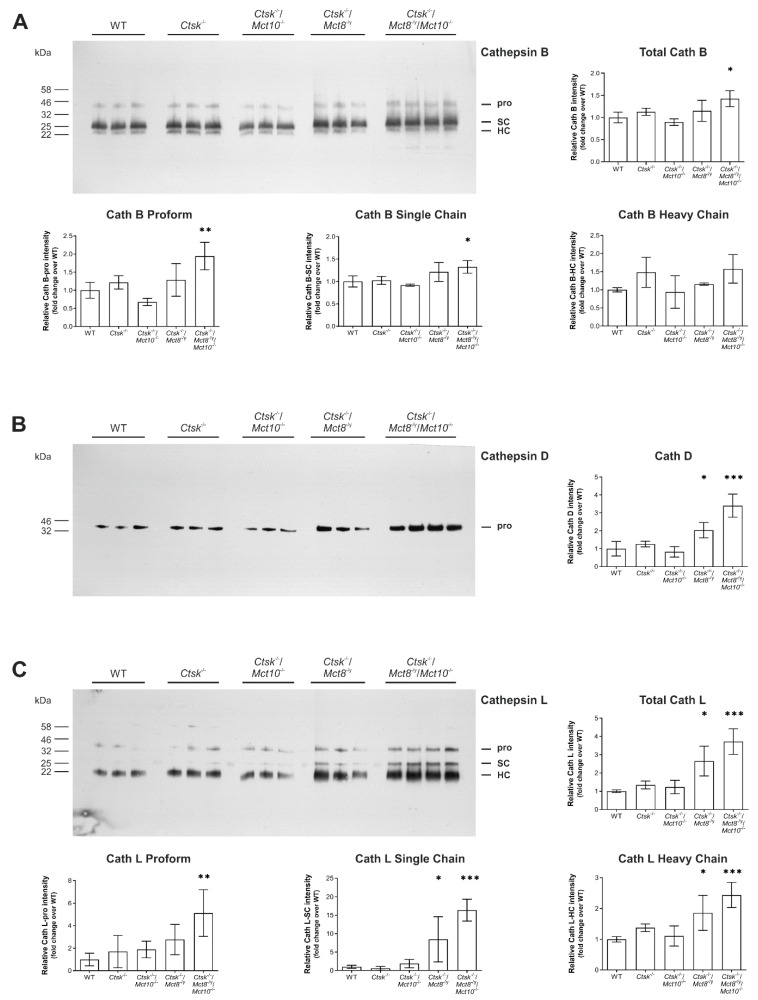
Maturation states of cathepsin B, D, and L remain unaltered in combined cathepsin K and TH transporter deficiency. Whole thyroid tissue lysates from WT, *Ctsk*^-/-^, *Ctsk*^-/-^/*Mct10*^-/-^, *Ctsk*^-/-^/*Mct8*^-/y^, and *Ctsk*^-/-^/*Mct8*^-/y^/*Mct10*^-/-^ mice were immunoblotted using antibodies specific for cathepsins B, D, or L, as indicated (**A**–**C**, respectively, left panels). Molecular mass markers are displayed in the left margins. Bands representing proform (pro), single-chain (SC), and heavy-chain (HC) of two-chain forms are indicated in the right margin of the immunoblots. The density of the resulting bands was normalized to total Ponceau-stained protein per lane. Bar graphs represent amounts of total cathepsins (**A**–**C**, right panels) and corresponding processed forms (**A**–**C**, bottom panels), respectively, as fold changes over WT. The total amounts of all investigated cathepsins were predominantly increased in *Ctsk*^-/-^/*Mct8*^-/y^/*Mct10*^-/-^ mice when compared to WT controls. The maturation states of cathepsins B and L did not differ in any genotype. Animals analyzed: *n* = 3–4 per genotype. Data is depicted as means ± SD. Levels of significance are indicated as * for *p* < 0.05, ** for *p* < 0.01, and *** for *p* < 0.001.

Cathepsins B, D, and L are present as proforms (pro) in the lumen of the endoplasmic reticulum (ER) and undergo activation upon arrival in endo-lysosomes through the proteolytic removal of the propeptides to become mature single- (SC) or two-chain forms, that consist of heavy (HC) and light chains (LC) held together by disulfide bonds [19]. The processing patterns depend on the presence of endosomal legumain or are mediated by autocatalysis [35]. However, there is no indication as of yet, whether single- or two-chain forms differ in proteolytic activity [36]. To investigate which cathepsin forms are present in increased amounts in *Ctsk*^-/-^/*Mct8*^-/y^ and *Ctsk*^-/-^/*Mct8*^-/y^/*Mct10*^-/-^ mice, thyroid tissue lysates of all genotypes were analyzed by immunoblotting using antibodies specific for cathepsin B, D or L (Figure 7A–C, upper left panels, respectively). Immunoblotting with cathepsin B- and L-specific antibodies revealed the presence of their expected molecular forms, i.e., pro, SC and HC of two-chain forms, while cathepsin D was detected only in its proform in all investigated genotypes. Quantification of the immuno-recognized bands by densitometry showed that the amount of thyroidal cathepsin B was exclusively enhanced in *Ctsk*^-/-^/*Mct8*^-/y^/*Mct10*^-/-^ mice, represented by a significant increase in the proform and single-chain cathepsin B levels (Figure 7A, upper right and bottom panels, respectively). However, both *Ctsk*^-/-^/*Mct8*^-/y^ and *Ctsk*^-/-^/*Mct8*^-/y^/*Mct10*^-/-^ mice showed an increase in the amounts of procathepsin D (Figure 7B, right panel) and of cathepsin L forms (Figure 7C, upper right panel). Although the single and heavy chain forms of cathepsin L were considerably enhanced in *Ctsk*^-/-^/*Mct8*^-/y^ and *Ctsk*^-/-^/*Mct8*^-/y^/*Mct10*^-/-^ thyroid lysates, a 5-fold increase in procathepsin L was observed only in the latter genotype (Figure 7C, bottom panels, respectively). While total levels of cathepsins B, D, and L appeared unaltered in *Ctsk*^-/-^ thyroid lysates (Figure 7A–C, upper right panels, respectively), the amounts of heavy chain cathepsin L were increased over WT controls, but this trend did not reach statistical significance in the Dunnett’s multiple comparison test (Figure 7C, bottom right panel). 

The results were in accordance with our previously published data on *Ctsk*^-/-^ mice at 10–15 months of age [20] and at 5–8 months of age [18]. Hence, we speculated whether the enhanced cathepsin L amounts in *Ctsk*^-/-^/*Mct8*^-/y^ and *Ctsk*^-/-^/*Mct8*^-/y^/*Mct10*^-/-^ mice are possibly caused by cathepsin K deficiency. However, closer inspection revealed that total amounts of cathepsin L were almost three to four-fold higher in *Ctsk*^-/-^/*Mct8*^-/y^ and *Ctsk*^-/-^/*Mct8*^-/y^/*Mct10*^-/-^ thyroids, respectively, than those of cathepsin K single knock-out mice, indicating that increased cathepsin L levels were also due to the additional Mct8 deficiency (Figure 7C, upper right panel). This observation is interesting in particular as it reveals redundant, compensatory regulation of cysteine cathepsins K and L [20] as well as counter-regulation of each protease in Mct8-deficiency ([18] and this study).

Taken together, we conclude that increased activities of cathepsins leading to increased proteolytic degradation of Tg in *Ctsk*^-/-^/*Mct8*^-/y^ and *Ctsk*^-/-^/*Mct8*^-/y^/*Mct10*^-/-^ thyroids, in particular, is a consequence of higher expression of cathepsin genes, resulting in enhanced translation of all forms of cathepsin proteins, whereas proteolytic maturation to distinct molecular forms is unaffected in either genotype. Moreover, the cathepsins are present as intact and stable proteins despite their enhanced presence in thyrocytes, because no obvious cathepsin degradation fragments were observed.

### 2.6. Enhanced Lysosomal Biogenesis Results in Higher Cathepsin Protein Amounts in Ctsk^-/-^/Mct8^-/y^ and Ctsk^-/-^/Mct8^-/y^/Mct10^-/-^ Mice

Since cathepsins B, D, and L are endo-lysosomal proteins with increased amounts in thyroid tissue of *Ctsk*^-/-^/*Mct8*^-/y^ and, more dramatically, in *Ctsk*^-/-^/*Mct8*^-/y^/*Mct10*^-/-^ mice, we next wanted to explore whether this is due to induction of lysosomal biogenesis. 

**Figure 8:**
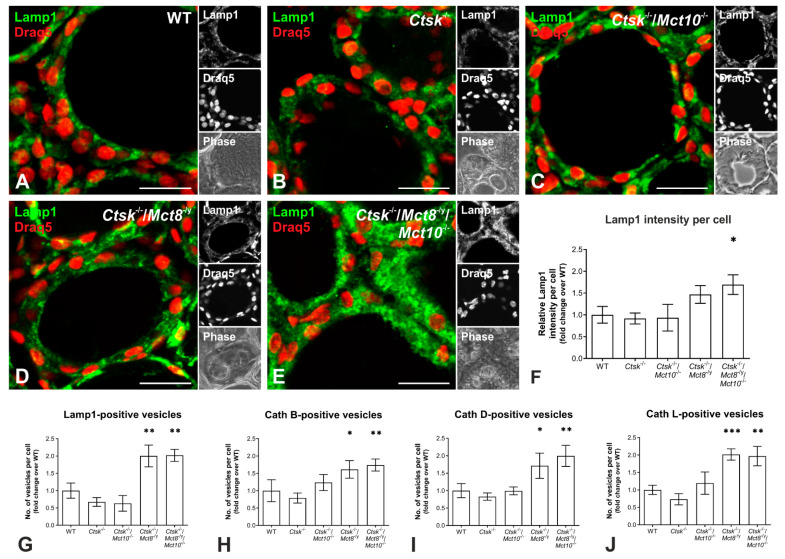
Assessing the induction of lysosomal biogenesis in mice lacking cathepsin K and TH transporters. Thyroid tissue sections from (**A**) WT, (**B**) *Ctsk*^-/-^, (**C**) *Ctsk*^-/-^/*Mct10*^-/-^, (**D**) *Ctsk*^-/-^/*Mct8*^-/y^, and (**E**) *Ctsk*^-/-^/*Mct8*^-/y^/*Mct10*^-/-^ mice were stained with antibodies against Lamp1 (green) and imaged by confocal laser scanning microscopy (**A**–**E**). As expected, Lamp1 signals were observed on vesicular membranes in all investigated genotypes. The intensity of Lamp1 staining (**F**) and the numbers of vesicles containing Lamp1 or cathepsins B, D or L (**G**–**J**, respectively) were determined using a Cell Profiler pipeline and normalized to the numbers of cells. Note that Lamp1 signals and the numbers of vesicles per cell were significantly increased in thyroid tissue of the triple-deficient genotype. Animals analyzed: *n* = 3 per genotype with 8–10 micrographs quantified per animal, respectively. Nuclei were counter-stained with Draq5^TM^ (red). Scale bars represent 20 µm. Data is depicted as fold changes over WT controls as means ± SD. Levels of significance are indicated as * for *p* < 0.05, ** for *p* < 0.01, and *** for *p* < 0.001.

Therefore, thyroid cryo-sections from WT, *Ctsk*^-/-^, *Ctsk*^-/-^/*Mct10*^-/-^, *Ctsk*^-/-^/*Mct8*^-/y^, and *Ctsk*^-/-^/*Mct8*^-/y^/*Mct10*^-/-^ were stained for Lamp1, a lysosomal membrane protein [37]. Immunofluorescence analysis showed that Lamp1 localized to vesicular compartments of thyrocytes in all inspected genotypes as expected (Figure 8A–E). However, the Lamp1 signal intensities appeared stronger in *Ctsk*^-/-^/*Mct8*^-/y^ and *Ctsk*^-/-^/*Mct8*^-/y^/*Mct10*^-/-^ thyroid tissue, and quantification demonstrated that the triple knock-out genotype displayed a two-fold increase in Lamp1 immunostaining intensity in comparison to WT controls (Figure 8F). 

To further inspect lysosomal biogenesis, the numbers of Lamp1- and cathepsin B-, D-, or L-positive vesicles were determined in immuno-stained cryo-sections of WT, *Ctsk*^-/-^, *Ctsk*^-/-^/*Mct10*^-/-^, *Ctsk*^-/-^/*Mct8*^-/y^, and *Ctsk*^-/-^/*Mct8*^-/y^/*Mct10*^-/-^ thyroid tissue using a Cell Profiler pipeline. The results showed that in *Ctsk*^-/-^/*Mct8*^-/y^ and *Ctsk*^-/-^/*Mct8*^-/y^/*Mct10*^-/-^ mice the per-cell numbers of Lamp1- and cathepsin-positive vesicles in thyroid tissue were significantly increased by almost 2-fold over WT controls (Figure 8G–J, respectively). This led us to believe that the observed increase in cathepsin amounts in the genotypes lacking Mct8, and particularly in the triple knock-out genotype (compare with Figure 7), is possibly the result of an overall increase in the expression of lysosomal proteins due to enhanced lysosomal biogenesis. 

**Figure 9:**
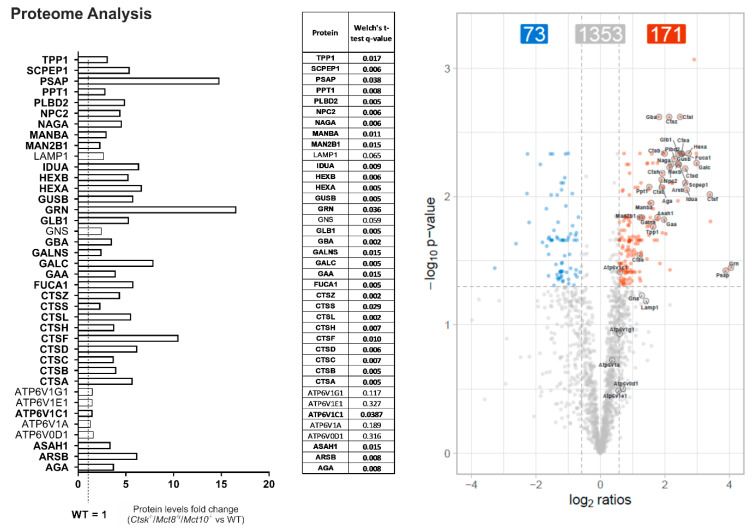
Proteome analysis of lysosomal constituents in thyroid tissue of *Ctsk*^-/-^/*Mct8*^-/y^/*Mct10*^-/-^ mice. Snap-frozen thyroid tissue from the triple-deficient murine model and WT controls were analyzed using LC-MS/MS. The bars depict fold changes of derived protein levels (means) of commonly known targets of lysosomal biogenesis and those that play a role in lysosomal function in comparison to WT controls (WT = 1, left panel). The *q*-values derived by Welch’s t-test indicated that the protein levels of 33 out of 39 lysosomal proteins were significantly increased in *Ctsk*^-/-^/*Mct8*^-/y^/*Mct10*^-/-^ vs. WT thyroid tissue (center panel). Bars and *q*-values for corresponding proteins that show significant differences are indicated in bold. Volcano plot of proteome data displaying the pattern of differential thyroid protein abundance for *Ctsk*^-/-^/*Mct8*^-/y^/*Mct10*^-/-^ mice relative to WT animals is shown (right panel). The *x*-axis indicates the log_2_ of the protein ratios in the comparison and the *y*-axis indicates the negative decadic logarithm of the *p*-values. Significantly differentially abundant proteins (*p* ≤ 0.05, fold change ≥ |1.5|) are highlighted as red or blue points, indicating proteins present in increased and decreased amounts in *Ctsk*^-/-^/*Mct8*^-/y^/*Mct10*^-/-^ mice relative to WT animals, respectively. Lysosomal proteins are explicitly labelled.

Because the alterations in cathepsin amounts, cysteine peptidase activities, and Lamp1-positive vesicles per cell were most pronounced in *Ctsk*^-/-^/*Mct8*^-/y^/*Mct10*^-/-^ mice, hinting to induced lysosomal biogenesis, snap-frozen thyroid tissue from these animals were subjected to proteome analyses for determination of levels of known lysosomal hydrolases and accessory proteins, lysosomal membrane proteins, and proteins important for lysosomal acidification. Interestingly, 33 out of 39 examined lysosomal proteins showed increased levels, with fold changes ranging from 3- to as high as 16-fold increase compared to WT controls, indeed providing further evidence that lysosomal biogenesis was induced in *Ctsk*^-/-^/*Mct8*^-/y^/*Mct10*^-/-^ mice (Figure 9).

### 2.7. Autophagy Is Induced in the Thyroid Glands of Ctsk^-/-^/Mct8^-/y^ and Ctsk^-/-^/Mct8^-/y^/Mct10^-/-^ Mice

**Figure 10:**
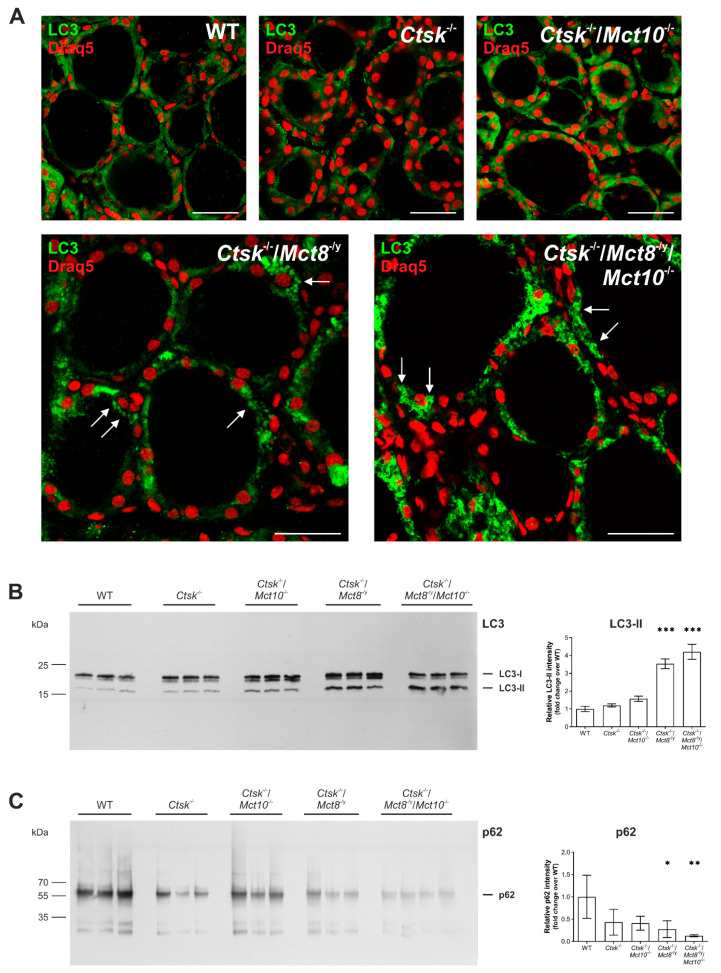
Autophagy in the thyroid glands of mice lacking cathepsin K and TH transporters. Mouse thyroid glands from the indicated genotypes were sectioned and stained with LC3-specific antibodies (**A**, green). Immunofluorescence analyses revealed diffuse staining patterns for LC3 in WT, *Ctsk*^-/-^, and *Ctsk*^-/-^/*Mct10*^-/-^, while LC3 signals were punctate and rather vesicular (arrows) in *Ctsk*^-/-^/*Mct8*^-/y^ and *Ctsk*^-/-^/*Mct8*^-/y^/*Mct10*^-/-^ thyroids (**A**). Nuclei were counter-stained with Draq5^TM^ (**A**, red). Scale bars represent 50 µm. Thyroid tissue lysates were separated on horizontal SDS-gels and immunoblotted for LC3 (**B**, left panel) or p62 (**C**, left panel). LC3-II and p62 band intensities were determined by densitometry and normalized to total Ponceau-stained protein per lane (**B** and **C**, right panels, respectively). Densitometry analyses confirmed that autophagy was induced in *Ctsk*^-/-^/*Mct8*^-/y^ and *Ctsk*^-/-^/*Mct8*^-/y^/*Mct10*^-/-^ mice. In both genotypes, the LC3-II signal was enhanced while the p62 signal was diminished in comparison to WT controls, corresponding to increased autophagosomal numbers and autophagic flux, respectively. Animals analyzed: *n* = 5–6 per genotype with 8–10 micrographs quantified per animal in A, respectively, and *n* = 3–4 per genotype in (**B**) and (**C**). Densitometry data is depicted as fold changes over WT as means ± SD. Levels of significance are indicated as * for *p* < 0.05, ** for *p* < 0.01, and *** for *p* < 0.001.

Because it is known that autophagy is one of the possible pathways leading to lysosomal biogenesis [38,39,40], we further asked whether this pathway is induced in *Ctsk*^-/-^/*Mct8*^-/y^ and *Ctsk*^-/-^/*Mct8*^-/y^/*Mct10*^-/-^ mouse thyrocytes. Thyroid cryo-sections from all genotypes were stained with an antibody specific for the autophagosomal marker microtubule-associated protein 1A/1B-light chain 3 (LC3) and imaged by confocal laser scanning microscopy. Micrographs were then analyzed to distinguish cytosolic from vesicular LC3 localization representative of non- or initiated autophagy, respectively (Figure 10). In WT, *Ctsk*^-/-^, and *Ctsk*^-/-^/*Mct10*^-/-^ tissue, LC3 was primarily detected in a diffuse pattern in the cytoplasm of thyrocytes (Figure 10A, upper panels, respectively). In *Ctsk*^-/-^/*Mct8*^-/y^ and *Ctsk*^-/-^/*Mct8*^-/y^/*Mct10*^-/-^ tissue, however, LC3 patterns changed to predominantly punctate structures (Figure 10A, lower panels, respectively). LC3-positive puncta were most frequently observed in *Ctsk*^-/-^/*Mct8*^-/y^/*Mct10*^-/-^ thyrocytes (Figure 10A, arrows in lower right panel). 

It is known that newly synthesized LC3 becomes processed to form cytosolic LC3-I, which later undergoes post-translational modification to form lipidated LC3-II that can integrate into intracellular membranes. While LC3-I localizes in the cytosol, LC3-II is associated with the inner and outer autophagosomal membranes during initiation of autophagy. Accordingly, the immunofluorescence data suggested that the anti-LC3-positive puncta that were prominent in *Ctsk*^-/-^/*Mct8*^-/y^ and *Ctsk*^-/-^/*Mct8*^-/y^/*Mct10*^-/-^ thyroid tissue represented an enhancement in vesicular integration of LC3-II, and thereby indicated autophagosome formation as a consequence of autophagy induction in these mice. To further confirm this interpretation, LC3 immunoblotting of thyroid tissue lysates was performed (Figure 10B, left panel). Owing to the higher hydrophobicity of the lipidated form, LC3-II migrates faster (17 kDa) on SDS-gels than LC3-I (19 kDa). Densitometry analysis of the immunoblots showed a significant increase in the intensity of LC3-II representative bands in *Ctsk*^-/-^/*Mct8*^-/y^ and *Ctsk*^-/-^/*Mct8*^-/y^/*Mct10*^-/-^ thyroid tissue lysates. While the former genotype displayed a 3-fold increase, the latter exhibited a 4-fold increase in LC3-II levels when compared to WT tissue lysates (Figure 10B, right panel). 

The enhancement in LC3-II levels can reflect two different scenarios, that is, (i) induced autophagosome formation or (ii) diminished autophagosome turnover (or autophagic flux) possibly due to an impairment in autophagosome-lysosome fusion and lysosomal activity, consequently leading to autophagosome accumulation. To examine the autophagic flux, we investigated the changes in the protein levels of p62 (SQSTM1/sequestosome 1), a protein that first interacts with LC3 and autophagic cargo, and subsequently delivers the cargo to autolysosomes for degradation. In this process, however, p62 is itself degraded when autophagy is induced and autophagic flux is high. Immunoblotting of thyroid tissue lysates with an antibody specific for p62 (Figure 10C, left panel) showed a significant reduction in its levels in *Ctsk*^-/-^/*Mct8*^-/y^ and *Ctsk*^-/-^/*Mct8*^-/y^/*Mct10*^-/-^ mice, indicating that autophagic activity is enhanced in these genotypes in comparison to WT or *Ctsk*^-/-^ and *Ctsk*^-/-^/*Mct10*^-/-^ animals (Figure 10C, right panel). 

In summary, the results indicated that while autophagy is unaltered in *Ctsk*^-/-^ and *Ctsk*^-/-^/*Mct10*^-/-^ thyrocytes, combined cathepsin K and Mct8 deficiency activates autophagy and maintains autophagic degradation, and these features became most prominent in the *Ctsk*^-/-^/*Mct8*^-/y^/*Mct10*^-/-^ triple-deficient murine model.

### 2.8. Intrathyroidal Iodine Levels Are Enhanced in Ctsk^-/-^/Mct8^-/y^ and Ctsk^-/-^/Mct8^-/y^/Mct10^-/-^ Mice

**Figure 11:**
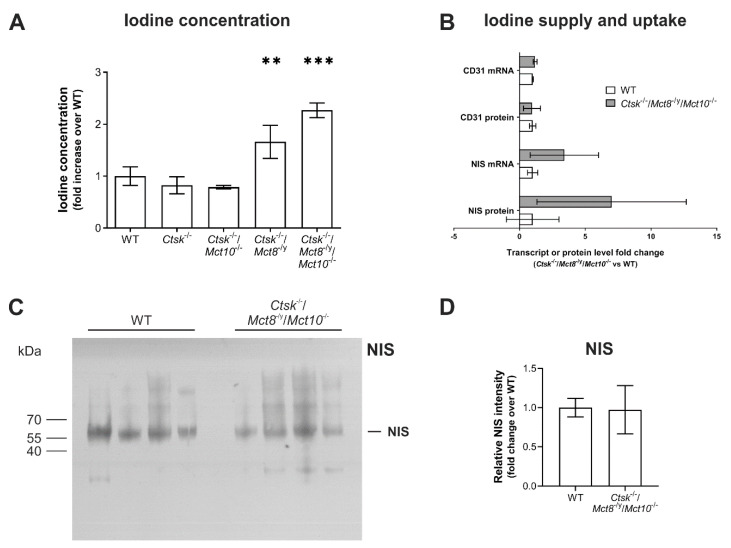
Intrathyroidal iodine levels in combined cathepsin K and TH transporter deficiency. Whole thyroid tissue lysates from WT, *Ctsk*^-/-^, *Ctsk*^-/-^/*Mct10*^-/-^, *Ctsk*^-/-^/*Mct8*^-/y^, and *Ctsk*^-/-^/*Mct8*^-/y^/*Mct10*^-/-^ mice were normalized to 1 µg of total protein and the SK reaction was performed to determine intrathyroidal iodine concentrations. Displayed is a bar graph representing iodine concentrations in all genotypes as fold changes over WT controls (**A**). Intrathyroidal iodine concentrations were significantly enhanced in *Ctsk*^-/-^/*Mct8*^-/y^, and more prominently in *Ctsk*^-/-^/*Mct8*^-/y^/*Mct10*^-/-^ mice. Snap-frozen thyroid tissue from *Ctsk*^-/-^/*Mct8*^-/y^/*Mct10*^-/-^ mice was further subjected to Omics analyses to study differential expression of genes and respective levels of proteins involved in iodine supply through the blood circulation (CD31) or by iodide uptake (NIS) (**B**), neither of which showed significant differences when compared to WT controls, while NIS mRNA and protein showed a trend toward increased levels. (**C**) Thyroid tissue lysates were separated on horizontal SDS-gels and immunoblotted for NIS (**C**), and the band intensities were determined by densitometry and normalized to total Ponceau-stained protein per lane (**D**). Molecular mass markers are displayed in the left margins (**C**). Bands representing NIS are indicated in the right margin of the immunoblots (**C**). Immunoblot and densitometry analyses confirmed that NIS amounts were not altered in *Ctsk*^-/-^/*Mct8*^-/y^/*Mct10*^-/-^ mice in comparison to WT controls (**C** and **D**). Densitometry data is depicted as fold changes over WT (**D**). Animals analyzed: *n* = 3–5 per genotype. Data is depicted as means ± SD. Levels of significance are indicated as ** for *p* < 0.01 and *** for *p* < 0.001.

Given the observations of enhanced Tg solubilization and degradation due to increased proteolytic activities of cathepsins by induction of autophagic lysosomal biogenesis in *Ctsk*^-/-^/*Mct8*^-/y^ and *Ctsk*^-/-^/*Mct8*^-/y^/*Mct10*^-/-^, we wondered whether the intrathyroidal TH states were altered. In addition to enhanced Tg utilization, these particular mice also lack TH transporters and would hence most possibly exhibit impaired TH release from the thyroid follicles, thereby causing intrathyroidal hyperthyroidism as previously described by us for Mct8 and/or Mct10-deficient mice [13,18]. TH are iodothyronines and a difference in their concentrations would be expected to result in a relative change in iodine concentrations in the tissue. Therefore, we performed the Sandell-Kolthoff (SK) reaction using thyroid tissue lysates from all genotypes to determine the intrathyroidal iodine concentrations normalized to protein. It was found that while the iodine concentration in thyroid tissue remained unchanged in *Ctsk*^-/-^ and *Ctsk*^-/-^/*Mct10*^-/-^ mice, the iodine concentrations in thyroid tissue of *Ctsk*^-/-^/*Mct8*^-/y^ and *Ctsk*^-/-^/*Mct8*^-/y^/*Mct10*^-/-^ animals were enhanced to almost 1.5- to 2-fold, respectively, over WT controls (Figure 11A). 

It should be noted that the thyroid gland accumulates iodine in high concentrations in the form of iodinated Tg compacted in an osmotically inert form within the thyroid follicle lumen [3,27]. Dietary iodine is supplied to the thyroid gland by the surrounding blood vessels and subsequently imported together with sodium via sodium-iodide symporter (NIS)-mediated transport across the basolateral plasma membrane of thyrocytes. Hence, changes in intrathyroidal iodine concentrations could be a consequence of any or all of the following, (i) increased iodine supply due to enhanced blood vessel support resulting from induced angio- or vasculogenesis, (ii) increased iodine accumulation due to an upregulation of sodium-iodide symporter (NIS), or (iii) increased Tg degradation and blocked export of TH such as observed in some of the genotypes (see above). Since the intrathyroidal iodine concentrations were altered most in the triple-deficient mouse model, snap-frozen thyroid tissue from these animals were subjected to transcriptome and proteome analyses to investigate differential expression of CD31 (a marker of blood vessels) and NIS (Figure 11B). Results showed that while transcript and protein levels of CD31 remained unchanged, a trend of increased NIS mRNA and protein levels was observed in *Ctsk*^-/-^/*Mct8*^-/y^/*Mct10*^-/-^ compared to the WT mice which, however, did not reach statistical significance. In order to further substantiate these data, immunoblotting with NIS-specific antibodies was performed using thyroid lysates of *Ctsk*^-/-^/*Mct8*^-/y^/*Mct10*^-/-^ animals in comparison to WT controls, revealing no difference in the intensity of NIS-representative bands (Figure 11C,D). These results implied that iodine supply and iodide trapping were only affected to a minor extent, if at all, in thyroid tissue of the *Ctsk*^-/-^/*Mct8*^-/y^/*Mct10*^-/-^ animals. Hence, the data suggested that increased iodine concentrations in thyroid tissue lysates of *Ctsk*^-/-^/*Mct8*^-/y^ and *Ctsk*^-/-^/*Mct8*^-/y^/*Mct10*^-/-^ mice are most likely reflecting TH accumulation as a result of increased Tg degradation and deficiency in exporting TH from the thyroid follicles into the blood circulation.

### 2.9. Accumulation of T3 in the Nuclei of Ctsk^-/-^/Mct8^-/y^ and Ctsk^-/-^/Mct8^-/y^/Mct10^-/-^ Thyrocytes

**Figure 12:**
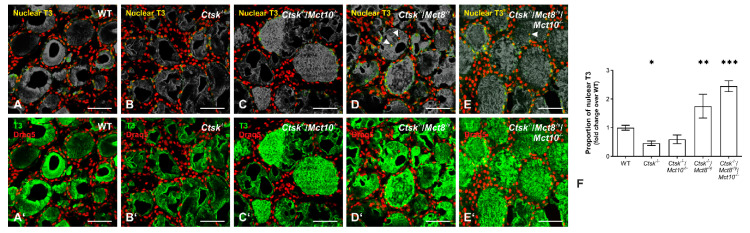
Nuclear T3 amounts in thyroids lacking cathepsin K and TH transporters. Cryo-sections of thyroid glands from WT, *Ctsk*^-/-^, *Ctsk*^-/-^/*Mct10*^-/-^, *Ctsk*^-/-^/*Mct8*^-/y^, and *Ctsk*^-/-^/*Mct8*^-/y^/*Mct10*^-/-^ mice were incubated with T3-specific antibodies (green) and analyzed by confocal laser scanning microscopy (A′–E′, respectively). T3 signals in the nuclei of thyrocytes (yellow) for all investigated genotypes are depicted upon desaturation of the T3-channel (**A**–**E**, respectively). Areas occupied by nuclear T3 over the total nuclear area was determined by a Cell Profiler-based pipeline representing an observer-unbiased approach, and the proportion of nuclear T3 signals are displayed as fold changes over WT controls (**F**). *Ctsk*^-/-^/*Mct8*^-/y^ and *Ctsk*^-/-^/*Mct8*^-/y^/*Mct10*^-/-^ mice show enhanced accumulation of T3 within the nuclei of thyroid epithelial cells, indicating thyrotoxicity. Note that a decrease in nuclear T3 was observed in *Ctsk*^-/-^ thyroids which feature enhanced Mct8-mediated TH export. Nuclei were counter-stained with Draq5^TM^ (red). The Mct8-deficient genotypes showed dead cells in follicle lumina with nuclear T3 (**D** and **E**, arrowheads). Scale bars represent 50 µm. Animals analyzed: *n* = 3 per genotype with 6–10 micrographs quantified per animal in A-E, respectively. Data is depicted as means ± SD. Levels of significance are indicated as * for *p* < 0.05, ** for *p* < 0.01, and *** for *p* < 0.001.

Thyrotoxic state of a tissue, or thyrotoxicity, refers to the excessive intracellular accumulation or retention of TH. The ‘toxic’ effects typically involve nuclear translocation of T3 and induction of gene transcription. In order to demonstrate intrathyroidal TH accumulation, we sought out to immunolabel WT, *Ctsk*^-/-^, *Ctsk*^-/-^/*Mct10*^-/-^, *Ctsk*^-/-^/*Mct8*^-/y^, and *Ctsk*^-/-^/*Mct8*^-/y^/*Mct10*^-/-^ mouse thyroid tissue sections with antibodies specific for T3 (Figure 12A–E,A′–E′, respectively). Because the TH are synthesized on the backbone of the precursor Tg, which is highly abundant in any thyroid follicle, Tg-bound T3 molecules provide epitopes for anti-T3 antibodies, thus leading to pronounced intraluminal immunostaining of the cryo-sections. Therefore, it becomes difficult to exclusively study the intracellular accumulation of biologically active T3 that is indicative of increased TH liberation by enhanced degradation of the prohormone Tg. To overcome this issue, the false-colored green channels (corresponding to T3 immunostaining) were desaturated, leaving behind only the stain that associates with the false-colored red channels (corresponding to Draq5™-stained nuclear DNA) to produce a yellow stain corresponding to T3 located in the nuclei (Figure 12A–E, respectively). Staining for T3 revealed an increase in its nuclear and cytosolic presence in thyroid epithelial cells of *Ctsk*^-/-^/*Mct8*^-/y^ and *Ctsk*^-/-^/*Mct8*^-/y^/*Mct10*^-/-^ mice in comparison to the WT littermates (Figure 12D,E). In some follicles, T3-positive nuclear remnants were observed over thyroid follicle lumina, indicative of a thyrotoxic state causing cell death in particular in the triple-deficient genotype (Figure 12D,E, arrowheads). In addition, using a Cell Profiler pipeline, we determined the genotypic differences in the amounts of T3 within the nuclei of thyrocytes (Figure 12F). Quantification of the area occupied by nuclear T3 over the area occupied by thyrocyte nuclei was determined in all genotypes under investigation by this observer-unbiased approach. Results revealed a striking increase of almost 2-fold over WT controls in the proportion of nuclear-associated T3 in *Ctsk*^-/-^/*Mct8*^-/y^ and *Ctsk*^-/-^/*Mct8*^-/y^/*Mct10*^-/-^ thyroid tissue. Interestingly, nuclear T3 amounts appeared reduced in comparison to WT controls in the thyrocytes of *Ctsk*^-/-^ and *Ctsk*^-/-^/*Mct10*^-/-^ mice, suggesting an intrathyroidal hypothyroid state in these animals (Figure 12). This latter observation could be explained by the increased presence of Mct8 in cathepsin K-deficiency [18], which could, in principle, cause enhanced TH export from thyroid follicles. 

Collectively, the data confirms the notion that lysosomal biogenesis is induced in thyroid epithelial cells. Mechanistically, this is brought about by autophagy, which results in increased Tg degradation in *Ctsk*^-/-^/*Mct8*^-/y^ and *Ctsk*^-/-^/*Mct8*^-/y^/*Mct10*^-/-^ mice. Enhanced Tg degradation leads to increased intrathyroidal TH accumulation because of the lack in Mct8-mediated TH export from thyroid follicles. Consequently, thyrotoxicity is caused due to intrathyroidal hyperthyroid states in *Ctsk*^-/-^/*Mct8*^-/y^ and *Ctsk*^-/-^/*Mct8*^-/y^/*Mct10*^-/-^ mice.

## 3. Discussion

Auto-regulatory mechanisms are important in coping with minor disturbances in thyroid gland function to maintain normal serum TH concentrations, thereby keeping up with TH demands of target organs. Cathepsins are essential for TH liberation prior to TH release from thyroid follicles into the blood circulation [3,9]. There is evidence that the lack of one cathepsins can be functionally compensated by the upregulation of a related cysteine cathepsin [19,20]. On the contrary, no such observations of one-to-one functional redundancy have been reported for TH transporters [11,13,41], which explains why TH transporter deficiency is in fact associated with altered thyroid phenotypes [11,13,18,41,42,43,44]. This study aimed to clarify whether TH transporters contribute to intrathyroidal auto-regulation of cathepsin-mediated Tg proteolysis. Thus, in this study, we investigated whether the lack of cathepsin K in Mct8 and/or Mct10 deficiencies would exacerbate the previously observed altered thyroid function phenotypes of *Mct8*^-/y^, *Mct10*^-/-^, and *Mct8*^-/y^/*Mct10*^-/-^ mice [18]. The data provides evidence that Mct8 plays a central part in non-classical auto-regulation of the thyroid gland, such that mice lacking both cathepsin K and Mct8, i.e., *Ctsk*^-/-^/*Mct8*^-/y^ and *Ctsk*^-/-^/*Mct8*^-/y^/*Mct10*^-/^^-^ degrade Tg continuously and excessively despite the inability of Mct8-deficient thyrocytes to properly export TH from thyroid follicles, eventually leading to intracellular TH accumulation. Regardless of the prominent role of Mct8 in thyroid auto-regulation, we cannot exclude the contribution of other TH transporters like the L-type amino acid transporter 2 (Lat2). Hence, future studies are required to identify all contributors to molecular sensing of intrathyroidal TH states. The results of the current study further unveil that induced lysosomal biogenesis through induction of autophagy—most possibly triggered by self-thyrotoxicity—is the molecular pathway that explains this uncontrolled Tg solubilization and degradation, which is mediated by enhanced amounts of cathepsins, resulting in a disturbed degradome in the triple-deficient mice.

### 3.1. Cathepsin K Deficient Mice Are a Suitable Model to Explore Auto-Regulatory Mechanisms of Thyroid Gland Function

Cysteine cathepsins belong to the peptidase sub-family C1A of family C1 of clan CA (papain-like peptidases) that play vital roles in numerous physiological and pathological processes [3,19,45,46]. Cathepsins in more general terms are categorized as aspartic, cysteine, and serine proteases according to the nucleophilic amino acids in the active sites of the enzymes [34]. Proteolysis of human and mouse Tg is mediated by cysteine cathepsins B, K, L, and S as well as aspartic cathepsin D [20,47,48,49,50]. Considering the importance of TH for proper functioning of central and peripheral target tissues as well as for the thyroid gland itself, it is both relevant and timely to better understand regulatory mechanisms that control the degree of proteolytic degradation of the precursor molecule Tg for subsequent TH liberation (for a perspective review, see [9]).

It is well understood that TRH from hypothalamic neurons causing TSH release from the anterior pituitary are both negatively regulated by circulating T3 and T4, respectively, to prevent unnecessary liberation and release of TH into the circulation [51]. Therefore, it is conceivable that murine models, in which amounts and/or activities of Tg-processing proteases are altered, might exhibit challenging thyroid phenotypes when compared to WT controls. Thus, as expected, mice deficient in cysteine cathepsins, *Ctsb*^-/-^ or *Ctsl*^-/-^, show accumulation of Tg in thyroid follicles due to its impaired proteolytic solubilization and degradation, while *Ctsl*^-/-^ and *Ctsk*^-/-^/*Ctsl*^-/-^ mice exhibit decreased serum concentrations of free T4 indicating hypothyroidism [20]. In addition, *Ctsb*^-/-^, *Ctsl*^-/-^, and *Ctsk*^-/-^/*Ctsl*^-/-^ thyrocytes show a significant enlargement of endo-lysosomes [20], which is reminiscent of disturbed lysosomal function such as seen in lysosomal storage disorders [52].

It is worth mentioning that the cysteine cathepsin K, in particular, carries the ability to directly liberate T4 from luminal Tg [49]. However, *Ctsk*^-/-^ mice show normal Tg degradation states when compared to WT animals, and are characterized by unaltered serum TH concentrations, albeit impaired luminal T4 liberation [20,21]. This interesting but unexpected observation was explained by upregulation of cathepsin L, thereby enabling functional compensation for the loss of cathepsin K [18,20]. Likewise, we have shown in the current study that aside from upregulation of mature cathepsin L, proteolytic maturation states of cathepsins B and D remained largely unchanged in thyroid tissue of *Ctsk*^-/-^ mice. Moreover, and albeit the observed increase in the proportion of DCG-04 reacted cysteine peptidases, gross Tg degradation in *Ctsk*^-/-^ thyroid glands was comparable to those of WT controls. Hence, cathepsin L carries out functional compensation for lacking cathepsin K, and this is enabled by transcriptional and post-translational means of regulation (this study and [20]). Reports indicating transcriptional regulation of cathepsin expression in FRTL-5 cells upon TSH stimulation, as seen for cathepsin B [53] and cathepsin S [54], provoked the question whether enhancement of cathepsin L amounts in the *Ctsk*^-/-^ thyroid gland is also regulated by TSH stimulated transcription of the *Ctsl* gene. Surprisingly, however, *Ctsk*^-/-^ mice feature normal serum TSH concentrations, hence eliminating the option of classical HPT axis regulation playing a role in increasing cathepsin L levels to functionally compensatory extents to keep gross Tg degradation unaltered in these animals [21]. Taken together, these findings make cathepsin K deficiency an excellent murine model to study thyroid auto-regulatory mechanisms in which altered Tg processing, rather than HPT axis regulation, keeps the serum TH status of *Ctsk*^-/-^ mice normal.

Cathepsin K deficiency is further presented with enhanced levels of the primary TH transporter Mct8 at the basolateral plasma membrane of *Ctsk*^-/-^ thyrocytes [18], which led us to propose the following putative scenario: (i) enhanced Mct8 in cathepsin K deficiency results in increased TH export from thyroid follicles, a process that might eventually result in transient intrathyroidal hypothyroid states, and (ii) upregulated cathepsin L-mediated Tg proteolysis in cathepsin K deficiency might be triggered as a result of sensing of the low intrathyroidal TH concentrations. In the current study, through immunohistochemical analysis of thyroid tissue stained with anti-T3 antibodies, we have indeed shown that the amounts of nuclear T3 are significantly reduced in *Ctsk*^-/-^ thyrocytes compared to WT controls, hinting at the proposed intrathyroidal hypothyroid state in cathepsin K deficiency. Keeping in mind that TH transporters like the Mct8 play important roles in TH transport, which is interconnected with cathepsin-mediated Tg proteolysis [18], it becomes all the more important to address what happens when Mct8 and/or Mct10 are additionally lacking in *Ctsk*^-/-^ mice. Our proposed scenario would predict that when Mct8 is missing, intrathyroidal hypothyroid states are no longer maintained, eventually leading to compensatory upregulation of cathepsins for enhanced Tg utilization. This in turn could result in intrathyroidal hyperthyroid states, eventually leading to self-thyrotoxicity, that would ultimately cause stress within the thyrocytes. Therefore, we asked in this study, whether *Ctsk*^-/-^/*Mct10*^-/-^, *Ctsk*^-/-^/*Mct8*^-/y^, or *Ctsk*^-/-^/*Mct8*^-/y^/*Mct10*^-/-^ mice show changes in thyroid functions and/or regulatory pathways induced by stress, that is, by initiation of autophagy.

### 3.2. Thyroid Gland Functionality Is Assessed Regarding TH Synthesis, TH Liberation and TH Release from Thyroid Follicles

In the current study, we have demonstrated that *Ctsk*^-/-^/*Mct8*^-/y^ and *Ctsk*^-/-^/*Mct8*^-/y^/*Mct10*^-/-^, but not *Ctsk*^-/-^/*Mct10*^-/-^ mice showed increased intrathyroidal iodine levels as well as enhanced nuclear T3 amounts, suggesting a state of self-thyrotoxicity. We reasoned that these observations cannot solely be explained by the absence of the primary TH transporter Mct8 at the basolateral plasma membrane of *Ctsk*^-/-^/*Mct8*^-/y^ and *Ctsk*^-/-^/*Mct8*^-/y^/*Mct10*^-/-^ thyrocytes, resulting in impaired TH export and intrathyroidal TH accumulation [11,13].

TH are synthesized on the backbone of Tg and liberated by proteolytic processing of the precursor. Therefore, thyroidal TH states can only be evaluated comprehensively by inspecting each and all of the following parameters (i) Tg biosynthesis, (ii) TH generation, (iii) Tg cross-linking, (iv) luminal Tg solubilization, and (v) endo-lysosomal Tg proteolysis. 

Comparative analyses of total Tg protein amounts with respect to WT controls showed no differences in either *Ctsk*^-/-^/*Mct8*^-/y^ or *Ctsk*^-/-^/*Mct8*^-/y^/*Mct10*^-/-^ mice, thereby eliminating “altered Tg biosynthesis” as a parameter that is responsible for the self-thyrotoxic states observed in these animals. Generation of Tg-bound TH occurs upon secretion into the thyroid follicle lumen and requires the iodination of tyrosine residues followed by intramolecular coupling to Tg-bound iodothyronines [55]. The sodium-iodide symporter NIS, localized at the basolateral plasma membrane of thyrocytes, mediates iodide uptake from the blood circulation [56,57]. Upon reaching the thyrocyte’s cytosol, iodide is transported across the apical plasma membrane by means of the chloride-iodide antiporter pendrin and the calcium-activated channel protein anoctamin 1, and the recently identified iodide transporter Slc26A7, resulting in its export into the extracellular follicle lumen [56,58,59,60]. Oxidation of iodide to iodine followed by its organification to form iodotyrosyl residues on Tg is mediated by apically located thyroid peroxidase (TPO) [61,62]. This process requires an oxidizing environment provided by the H_2_O_2_ generating system consisting of two dual oxidases (DUOX1 and DUOX2) and their activating maturation factors (DUOXA1 and DUOXA2) [63]. In the current study, we found that iodide supply and uptake, as determined by the amounts of CD31, as proxy for blood vessels, and NIS, respectively, were comparable between *Ctsk*^-/-^/*Mct8*^-/y^/*Mct10*^-/-^ and WT control transcriptomes and proteomes of thyroid tissue. A trend toward increased protein amounts of NIS was observed, which might hint toward enhanced iodide accumulation within thyrocytes of *Ctsk*^-/-^/*Mct8*^-/y^/*Mct10*^-/-^ animals. However, this is unlikely playing a significant role as statistical significance was not reached and immunoblotting demonstrated no change in protein amounts or stability either. The mRNA levels of TPO, pendrin, anoctamin 1, DUOXs, and DUOXAs remained unchanged compared to WT, while Slc26A7 was upregulated (unpublished data), thereby suggesting that at least in the triple-deficient genotype “TH generation” was only marginally altered if at all. Hence, other mechanisms must explain why in particular the *Ctsk*^-/-^/*Mct8*^-/y^/*Mct10*^-/-^ murine model showed the most altered intrathyroidal thyroid states.

Tg in transit and iodinated Tg undergo compaction for luminal storage in osmotically inert and covalently cross-linked form by processes to which several enzymes contribute, including the disulfide bridge-forming protein disulfide isomerase [3,27,28]. Since the densities of Tg bands representing multimers and dimers were comparable between all investigated genotypes in non-reducing SDS-PAGE analyses, we conclude that “Tg crosslinking” remains unaffected in *Ctsk*^-/-^/*Mct8*^-/y^ and *Ctsk*^-/-^/*Mct8*^-/y^/*Mct10*^-/-^ mice. Thus, altered cross-linking is not the cause for the altered Tg degradation states seen in these animals.

Solubilization of Tg occurs from the intraluminally stored cross-linked multimeric forms before and upon endocytic reuptake, respectively, and results in subsequent Tg degradation for TH liberation [3,9,50]. Previously, we reported that cathepsins B and L are mainly responsible for the solubilization of Tg from its cross-linked forms [20]. As expected from compensatory upregulation of cathepsin L and our previous observations, significantly more cathepsins B and L of *Ctsk*^-/-^/*Mct8*^-/y^ and *Ctsk*^-/-^/*Mct8*^-/y^/*Mct10*^-/-^ thyroid tissue, in comparison to WT controls, explain increased “Tg solubilization”. This was represented by increased amounts of Tg fragments at the expense of dimeric and monomeric Tg forms which were reduced in *Ctsk*^-/-^/*Mct8*^-/y^ and *Ctsk*^-/-^/*Mct8*^-/y^/*Mct10*^-/-^ thyroid tissue. Moreover, increased endo-lysosomal cathepsin B, D, and L signals of *Ctsk*^-/-^/*Mct8*^-/y^ and *Ctsk*^-/-^/*Mct8*^-/y^/*Mct10*^-/-^ thyroid tissue in comparison to WT controls correlated well to the enhanced density of Tg fragments-representative bands, indicating increased “endo-lysosomal Tg proteolysis” in these mice. Collectively, we conclude that the absence of Mct8, in particular, in cathepsin K-deficient background affects the molecular pathways of Tg solubilization and degradation rather than Tg compaction and TH generation in a mechanism that enhances the amounts of active enzymes that mediate proteolytic Tg processing.

Intriguingly, differences in gross Tg degradation states were observed between *Ctsk*^-/-^/*Mct8*^-/y^ and *Ctsk*^-/-^/*Mct8*^-/y^/*Mct10*^-/-^ mice, because the decline in Tg dimer and monomer bands and the concomitant increase in Tg fragments were more pronounced in the triple-deficient mice. This indicates that, although both genotypes lack Mct8, the extent of cathepsin-mediated Tg proteolysis is most increased in *Ctsk*^-/-^/*Mct8*^-/y^/*Mct10*^-/-^ mice, which correlates well with the most enhanced protein amounts and proteolytic activities of Tg-processing enzymes in the triple-deficient animals. Obviously, a possible role of Mct10 deficiency can be ruled out in explaining cathepsin upregulation, because *Ctsk*^-/-^/*Mct10*^-/-^ mice feature cathepsin protein levels and cysteine peptidase activities comparable to WT thyroid tissue.

To understand whether differences in Tg degradation states arose from a misbalance of proteolytic versus anti-proteolytic factors in the thyroid of the investigated mice, we inspected the presence of endogenous cysteine protease inhibitors, the cystatins. Surprisingly, total cystatin C and D amounts were enhanced in *Ctsk*^-/-^/*Mct8*^-/y^ and even more so in *Ctsk*^-/-^/*Mct8*^-/y^/*Mct10*^-/-^ thyroid glands. Hence, that data did not explain why the expression levels and proteolytic activities of thyroidal cysteine cathepsins differed between *Ctsk*^-/-^/*Mct8*^-/y^ and *Ctsk*^-/-^/*Mct8*^-/y^/*Mct10*^-/-^ mice, and hinted to an overall induced lysosomal biogenesis.

### 3.3. Lysosomal Biogenesis via the Induction of Autophagy Is the Cause for the Unusual Intrathyroidal Hyperthyroid States Observed upon Combined Cathepsin K and Mct8 Deficiency

Our results revealed that apart from the increased cathepsin and cystatin amounts, thyroid tissue from mice lacking both cathepsin K and Mct8 also showed enhanced Lamp1 immuno-positive signals. In addition, endocytic compartments harboring cathepsins and Lamp1 were increased in numbers on a per-cell basis, thus, providing evidence for the induction of lysosomal biogenesis in the thyroid glands of *Ctsk*^-/-^/*Mct8*^-/y^ and *Ctsk*^-/-^/*Mct8*^-/y^/*Mct10*^-/-^ mice. It should be noted, however, that lysosomal biogenesis was more pronounced in the *Ctsk*^-/-^/*Mct8*^-/y^/*Mct10*^-/-^ than in *Ctsk*^-/-^/*Mct8*^-/y^ thyroids (see also above regarding Tg degradation states). Hence, the question arose, by which molecular mechanism lysosomal biogenesis was achieved in these genotypes.

Biogenesis of lysosomes is a process tightly regulated by the transcription factor EB (TFEB), in that TFEB translocates to the nucleus upon activation by desphosphorylation, and binds to the Coordinated Lysosomal Expression and Regulation (CLEAR) motif [64]. Considering that both, cathepsin- and cystatin-encoding genes, contain the CLEAR element [64], induced lysosomal biogenesis in the thyroid glands of *Ctsk*^-/-^/*Mct8*^-/y^ and *Ctsk*^-/-^/*Mct8*^-/y^/*Mct10*^-/-^ mice might explain their increased levels compared to WT controls. Furthermore, TFEB orchestrates the autophagy pathway by inducing biogenesis of autophagosomes and promoting autophagosome-lysosome fusion, thereby enhancing autophagic flux [39]. Autophagy involves proteolytic degradation of cellular macromolecules for turnover, and, hence, relies on the availability and activity of lysosomal proteases. Thus, TFEB activation links two important cellular processes, autophagy and lysosomal biogenesis [39].

The results of this study reveal that the induction of autophagy is accompanied by enhanced biogenesis of cathepsins, cystatins, and Lamp1 in *Ctsk*^-/-^/*Mct8*^-/y^ and *Ctsk*^-/-^/*Mct8*^-/y^/*Mct10*^-/-^ mice such, that the latter genotype exhibits both a higher degree of autophagosome formation (shown by the increase in LC3-II) as well as autophagic flux (shown by the decrease in p62 levels), and thereby portrays higher lysosomal biogenesis than the former genotype. However, this opens yet another question, that is, what triggers autophagy in mouse thyroids that lack Mct8 in a cathepsin K-deficient background? In the current study, we report that *Ctsk*^-/-^/*Mct8*^-/y^ and *Ctsk*^-/-^/*Mct8*^-/y^/*Mct10*^-/-^ thyroid glands exhibit enhanced intrathyroidal TH accumulation. It is well known that tissues deemed as “hyperthyroid” display enhanced levels of reactive oxygen species (ROS) [65]. Interestingly, it has also been found that lysosomal activity and autophagy can be induced by TH, particularly due to T3-induced oxidative stress in TH-target organs such as the liver and the skeletal muscle via TH-mediated AMP-activated protein kinase (AMPK) and Unc-51 related kinase (ULK1) activation [66,67,68]. It was further reported that TH activate TFEB, by inhibiting the mammalian target of rapamycin complex 1 (mTORC1), and thus induce lysosomal biogenesis in TH target tissues [69]. Our data on intrathyroidal iodine levels determined by the SK method and morphological analysis of thyroidal T3 in nuclear compartments shows that both parameters were predominantly upregulated in *Ctsk*^-/-^/*Mct8*^-/y^/*Mct10*^-/-^ in comparison to *Ctsk*^-/-^/*Mct8*^-/y^ mice. Accordingly, autophagy as well as lysosomal biogenesis was most pronounced in the former genotype. We conclude that intrathyroidal thyrotoxic states cause stress and ROS-activated autophagy resulting in enhanced lysosomal biogenesis specifically in the thyroid glands of *Ctsk*^-/-^/*Mct8*^-/y^/*Mct10*^-/-^ mice.

It should be noted that there might be further causes of stress-induced autophagy triggered in the murine thyroid gland, e.g., by misfolding of Tg resulting in its accumulation in the ER causing an unfolded protein response [70,71]. However, our data do not provide indications for such a mechanism because Tg protein amounts were not altered.

### 3.4. Perspectives in Light of Fast, Non-Genomic TH Actions

Recently, our understanding of TH action has been challenged and was deepened by the notion of fast, non-genomic actions of T3 and T4 that complement the well-known slow genomic effects of T3 [72]. Yet, no evidence or detailed investigation on such effects within the thyroid gland itself have been reported. In this context, it is worth mentioning that several studies provided evidence for the non-genomic effects of T3 on intracellular Ca^2+^ mobilization [73,74,75]. Recent studies have further highlighted the role of cytosolic Ca^2+^ levels in regulating autophagic activity [76,77]. In conclusion, we propose that the enhanced intracellular TH concentrations in Mct8-deficient thyroid glands cannot only result in self-thyrotoxicity by genomic means, but enhanced cytosolic TH may additionally cause ROS-induced stress and/or affect cytosolic Ca^2+^ levels, eventually resulting in autophagy induction for subsequent upregulation of endo-lysosomal proteins. Such pathways would encompass upregulation of the cathepsins, and their increased proteolytic activities would lead to enhanced Tg degradation for TH liberation, resulting in further enhanced self-thyrotoxicity and more stress. On top of this already vicious cycle, the increased accumulation of TH, due to impaired export, can further trigger autophagy and lysosomal biogenesis by fast non-genomic TH action, hence, counterintuitively further advancing Tg degradation. The results of the current study have set the stage for the required further in-depth examination of this hypothesis in mice with defects in non-genomic TH action.

## 4. Materials and Methods

### 4.1. Animals

The murine models used in the present study include *Ctsk*^-/-^, *Ctsk*^-/-^/*Mct10*^-/-^, *Ctsk*^-/-^/*Mct8*^-/y^, *Ctsk*^-/-^/*Mct8*^-/y^/*Mct10*^-/-^ and WT C57Bl/6J male mice at 5 to 8 months of age. The founder *Ctsk*^-/-^ mice were generated and kindly provided by Dr. Paul Saftig (Christian-Albrechts-Universität zu Kiel, Kiel, Germany), *Mct8*^-/y^ mice were provided by U.S. and E.K.W., *Mct10*^-/-^ mice were generated by F.V., and *Mct8*^-/y^/*Mct10*^-/-^ mice were provided by H.H. The mice were housed in the transgenic animal facility of Jacobs University Bremen (Bremen, Germany) by maintaining standardized conditions of light and dark cycles (12 h/12 h) and water and food *ad libitum*. *Ctsk*^-/-^/*Mct10*^-/-^, *Ctsk*^-/-^/*Mct8*^-/y^, and *Ctsk*^-/-^/*Mct8*^-/y^/*Mct10*^-/-^ were generated by crossbreeding sexually mature mice at the age of 6 to 8 weeks to generate homozygous littermates of the genotypes analyzed in the current study. Genotyping of mice was performed in accordance with previously described protocols [78] for *Ctsk*, [43,79] for *Mct8*, and [12] for *Mct10*; [18,21]. 

### 4.2. Tissue Sampling and Cryosectioning

Mice were euthanized by CO_2_ inhalation and vertical cuts were made through the skin to expose the abdominal and thoracic cavities. Blood was collected from the right ventricle of the heart using a 1 mL syringe with a 20-gauge needle. Perfusion was done by cutting the abdominal aorta to ensure blood outflow and injecting a solution of 0.9% NaCl supplemented with 200 IU heparin (Braun Melsungen AG, Melsungen, Germany) through the left ventricle of the heart, until the liver and kidneys appeared pale. The thyroid gland still attached to the trachea was dissected and fixed overnight at 4 °C in 4% paraformaldehyde in 200 mM HEPES, pH 7.4. Following fixation, thyroid glands were embedded in Tissue Freezing Medium (Jung, through Leica Microsystems, Nussloch, Germany) and incubated overnight at 4 °C for cryo-preservation. Then, thyroid tissue was frozen in the gas phase of liquid nitrogen and stored at −80 °C. Prior sectioning, thyroid tissue was placed inside a cryostat (Leica CM1900, Leica Microsystems) for 1 to 2 h to become acclimatized to −20 °C, cut into 5 µm thick transverse sections, mounted onto microscopic slides, and stored at −20 °C until further use for immunohistochemistry studies. For protein biochemistry analyses, thyroid lobes were separated from the trachea and snap-frozen in liquid nitrogen and stored at −80 °C until subsequent use for tissue lysate preparation.

### 4.3. Indirect Immunofluorescence

Staining of tissue sections for immunohistochemistry was performed as described [18,21,33] with the following specifications. Microscopic slides with thyroid tissue sections that were washed with calcium- and magnesium-free PBS (CMF-PBS) to remove residual embedding medium and blocked in 3% bovine serum albumin (BSA; Carl Roth GmbH, Karlsruhe, Germany) in CMF-PBS for 1 h at 37 °C, were incubated with primary antibodies diluted in 0.1% BSA in CMF-PBS in a moisturized chamber overnight at 4 °C. Specific primary antibodies and respective dilutions that were used in this study are listed in Table 1. Unbound primary antibodies were removed by washing the slides in 0.1% BSA in CMF-PBS, three times for 5 min each, at room temperature. Secondary antibodies and counter-stains were diluted together in 0.1% BSA in CMF-PBS and incubated for 1 h at 37 °C. Secondary antibodies used in this study included Alexa Fluor 488-conjugated goat anti-mouse IgG, goat anti-rabbit IgG, and rabbit anti-goat IgG (1:200; Molecular Probes, Karlsruhe, Germany). Draq5^TM^ (10 µM; BioStatus Limited, Shepshed, Leicestershire, UK) served as a counter-stain to visualize nuclear DNA.

To stain the cytoplasm of thyroid epithelial cells, tissue sections were incubated with HCS CellMask^TM^ Orange for 1 h at 37 °C (1:1000; #H32713, Molecular Probes, Eugene, OR, USA). In order to visualize glycosylated molecules in the thyroid tissue, slides were treated with biotinylated lectin concanavalin A from *Canavalia ensiformis* (ConA; #C2272, Sigma-Aldrich, Munich, Germany) at a final concentration of 10 µg/mL for 30 min at 4 °C, followed by incubation with Alexa Fluor 546-conjugated streptavidin (1:200; #S-11225, Molecular Probes). Slides were then washed in CMF-PBS and deionized water and finally mounted with embedding medium consisting of 33% glycerol and 14% Mowiol (Elvanol) in 200 mM Tris-HCl, pH 8.5 (Hoechst AG, Frankfurt, Germany) for microscopic analyses.

### 4.4. Image Acquisition and Automated Image Analysis

Stained thyroid tissue sections were imaged using a Zeiss LSM 510 META laser scanning microscope equipped with argon and helium-neon lasers (Carl Zeiss Microscopy GmbH, Oberkochen, Germany). Images were taken with a pinhole opening of 1 Airy unit and at a resolution of 1024 × 1024 pixels. Micrographs were analyzed using LSM 5 software (version 3.2; Carl Zeiss Microscopy GmbH) and stored in TIFF format. To ensure reliable quantification of fluorescence intensities, tissue sections were imaged at the same detector gain settings so that measurements from different murine models could be compared. The number of biological replicates per genotype used in this study and the number of thyroid follicles or micrographs analyzed are mentioned in the respective figure legends.

**Figure 13:**
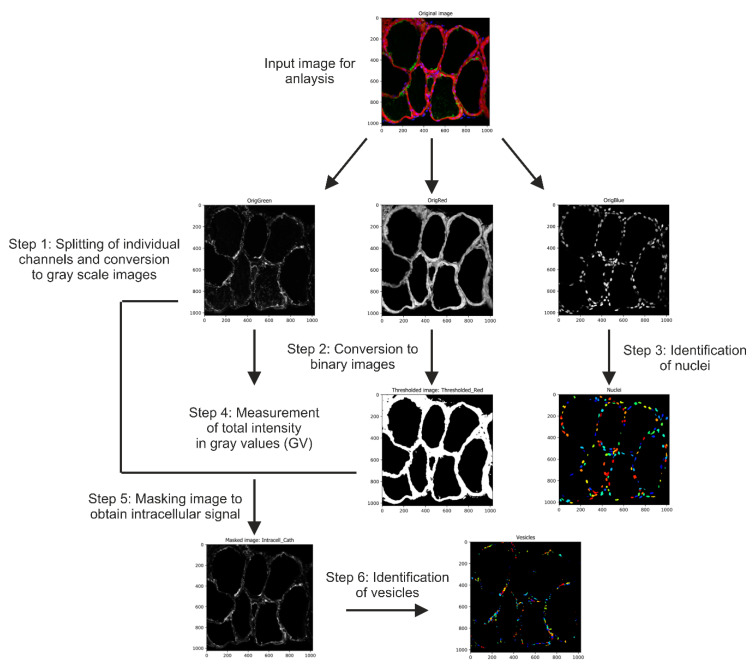
Automated image analysis using a Cell Profiler pipeline. Schematic diagram outlining the modules used for quantifying cathepsin amounts and numbers of cathepsin-positive vesicles within thyrocytes. As an example, a thyroid tissue section stained with cathepsin B antibody is shown. The input image contains three channels, namely, cathepsin (green), CMO stain for cell cytoplasm (red), and Draq5^TM^ counter-stain for nuclei (blue). (1) The input image was split into individual channels and converted to gray scale output images OrigRed, OrigGreen, and OrigBlue, respectively. (2) The OrigRed image representative of the CMO cytoplasmic staining was converted to a binary image after applying a threshold to eliminate any unspecific signal in the lumen. (3) The OrigBlue image was used to identify the nuclei. (4) To obtain total cathepsin intensity, the signal from the OrigGreen image was measured. (5) OrigRed was used to mask OrigGreen to exclusively detect immuno-positive signals in the epithelium (‘IntraCellular_Cath’). (6) IntraCellular_Cath was used to identify vesicles which were counted. Nuclear counts (step 3) were used to normalize as indicated in illustrations of the respective analyses.

Fluorescence measurements were done using an open-source automated image analysis software, Cell Profiler (version 3.1.9) [81], available from the Broad Institute at www.cellprofiler.org. In order to quantify fluorescence intensities and to count the numbers of Lamp1- or cathepsin-positive vesicles, a pipeline combining different image-analysis modules was developed (Figure 13). The arrangement of each module and the details describing its function in the pipeline is detailed sequentially: (i) The module ‘ColorToGray’ splits the input image into respective channels: Green (Lamp1 or cathepsins B, D, or L), Red (CMO), and Blue (Draq5). Further, the resulting split channels are converted to gray scale output images and are named as OrigGreen, OrigRed, and OrigBlue, respectively. (ii) The ‘ApplyThreshold’ module converts the OrigRed image to a binary image (black and white image) by applying a manual threshold value. A pixel intensity below this value is set ‘black’, while that above this value is set ‘white’. The output image was named as ‘Thresholded_Red’. The manual threshold value was determined depending on the CMO staining, such that the signal is restricted to the epithelium, and does include signal from the apical pericellular space or from within the thyroid follicle lumen. (iii) ‘IdentifyPrimaryObjects’ module aims to measure the total number of thyroid cells per image. It uses the OrigBlue image and identifies nuclei by considering a user-defined diameter range. This range depends on the magnification of the input image as well as its resolution. In the current pipeline, the minimum and the maximum values were set to 10 and 50 pixels, respectively. (iv) The module ‘MeasureImageIntensity’ quantifies the overall intensity of a particular channel in a given image. This module was used to measure the fluorescence intensity of the OrigGreen image, and hence determines the total immunofluorescence signal. (v) To specifically identify the intracellular signal, excluding the follicle lumen, the module ‘MaskImage’ was applied. Herein, the OrigGreen image is masked by the ‘Thresholded_Red’ image to produce the output image ‘IntraCellular_Cath’ that exclusively consists of thyroid epithelia identified from all follicles of the input image. (vi) To enumerate cathepsin- or Lamp1-positive vesicles, another ‘IdentifyPrimaryObjects’ module was introduced to identify vesicles within the image ‘IntraCellular_Cath’. This was done by manually setting a defined range of 5 to 50-pixel units to exclusively recognize vesicles in thyroid epithelial cells. These values were adjusted according to the image resolution. The fluorescence intensities of cystatin C, cystatin D, ConA, or Lamp1 labeling were quantified by measuring the respective image intensity of the green channel, OrigGreen, as described above in step iv. The signal measurements and vesicle counts were normalized to the number of cells that were determined from Draq5™ staining, and counted in step iii of the pipeline, and hence aided comparative analyses between different genotypes.

To investigate the proportion of nuclear T3 in different genotypes, merged channel micrographs with T3-staining visualized in green and nuclear DNA visualized in red were analyzed using another Cell Profiler-based pipeline. Following the ‘ColorToGray’ module to split the respective channels, OrigGreen and OrigRed, the nuclei were identified by applying the ‘IdentifyPrimaryObjects’ module as described above. Next, to exclusively study the T3 signal overlapping the signal from counter-staining of cell nuclei, the module ‘MaskImage’ was used with OrigGreen as the input image and OrigRed as the masking image, and the output image hence obtained was called ‘MaskedT3’. Using the module ‘MeasureImageAreaOccupied’, the area occupied by ‘MaskedT3’ was determined and normalized to the area occupied by nuclei, and thereby the genotypic differences in the amounts of immuno-stained nuclear T3 were examined.

### 4.5. Tissue Lysate Preparation, SDS-PAGE and Immunoblotting

Snap-frozen lobes of thyroid glands were lysed in ice-cold PBS, pH 7.4 supplemented with 0.5% Triton X-100 and protease inhibitors (0.02 M EDTA, 10 µM E64, 1 µM pepstatin A, and 2 ng/mL aprotinin) using a hand-held homogenizer, and further incubated on ice for 1 h. To study active cysteine peptidases in the thyroid lysates, biotin-conjugated activity-based probe DCG-04 [82] was added at a final concentration of 5 µM to the lysis buffer and the inclusion of protease inhibitors was omitted. Lysates were cleared by centrifugation at 16,000× *g* for 10 min at 4 °C, and stored at −20 °C. Protein concentrations were quantified using the Neuhoff assay as described [83]. Samples for immunodetection were prepared by normalizing tissue lysates to equal protein amounts, i.e., 0.6 µg/µL protein per sample was used and 15 µg were loaded per lane. Samples were denatured in sample buffer (50 mM Tris-HCl (pH 7.6), 2.5% sodium dodecyl sulfate (SDS), 125 mM dithiothreitol, and 4 μM bromophenol blue) for 5 min at 95 °C. Then, proteins were separated on commercially available horizontal SDS Gradient 8–18 ExcelGel™ gels (GE Healthcare, Uppsala, Sweden). PageRuler-prestained protein ladder (#P7712S, NEB, Frankfurt am Main, Germany or #26616, Thermo Fisher Scientific, Schwerte, Germany) was loaded on the horizontal gels to determine the molecular masses of separated proteins in the samples. Electrophoresis was carried out at 300 V and 50 mA by placing the horizontal gels on a cooling plate (Cooler Multitemp III, GE Healthcare), using paraffin oil, along with ExcelGel^TM^ SDS buffer strips in a horizontal gel electrophoresis chamber Multiphor II (GE Healthcare). Where indicated, samples were prepared in lysis buffer without DTT to examine proteins separated under non-reducing conditions. Next, proteins were transferred from the horizontal gels onto nitrocellulose membranes (Carl Roth) by semi-dry blotting in a Novoblot Western Blotting chamber (GE Healthcare) for 1 h at 30 V and 216 mA. Membranes were incubated for 10 min with Ponceau S solution (#A2395, AppliChem, Darmstadt, Germany) to visualize total protein per lane (Supplementary Figures S1–S6), which upon scanning served as a loading control for normalization. Next, membranes were destained with 0.1% NaOH and blocked using 5% blotting-grade milk powder in PBS containing 0.3% Tween-20 (PBS-T) for 1 h at room temperature. Then, membranes were incubated in primary antibodies diluted in PBS-T for 16 h at 4 °C. Primary antibodies used for immunoblotting are detailed in Table 1. Following washing with PBS-T for 30 min, membranes were incubated in horseradish peroxidase (HRP)-conjugated goat anti-rabbit IgG (H + L), goat anti-mouse IgG (H + L), and rabbit anti-goat IgG (H + L) secondary antibodies diluted (1:5000) in PBS-T supplemented with 2.5% milk powder for 1 h at 25 °C. To detect cysteine cathepsins labelled with DCG-04, membranes were blocked in 3% BSA and incubated in HRP-conjugated streptavidin (1:5000) for 1 h at room temperature, while lysates prepared without addition of DCG-04 were used as controls. Subsequently, membranes were washed once again in PBS-T for 30 min, and the immunoreactive proteins were visualized by enhanced chemiluminescence after incubating the membranes with ECL Western blotting substrate for 3 min and exposing them onto CL-XPosure^TM^ films (Pierce via Thermo Fisher Scientific). Exposed films were scanned using a transmitted-light scanner (Desk Scan II version 2.9, Hewlett-Packard Co., Palo Alto, CA, USA). Band intensities of target proteins and total protein per lane, obtained by immunoblotting and Ponceau S staining, respectively, were measured by densitometry analysis using Image Studio Lite version 5.2 (LI-COR Biosciences GmbH, Bad Homburg, Germany).

### 4.6. Iodine Quantification in Thyroid Tissue Lysates

Iodine concentrations in mouse thyroid lysates were determined by using a modified protocol based on the Sandell-Kolthoff (SK) reaction, a method that is traditionally used to quantify urinary iodine levels to study the prevalence of iodine deficiency disorders [84,85]. The SK reaction is a two-step process that involves, (i) the conversion of iodide (I^−^) in iodinated substances, such as proteins, to iodine (I_2_) by oxidative digestion using chemicals such as chloric acid and ammonium persulfate, and (ii) sequential reduction of ceric ammonium sulfate (yellow) to the cerous form (colorless) in the presence of arsenious acid, as given in Equation (1). The rate of disappearance of the yellow Ce^4+^ solution is proportional to the I_2_ concentration in the sample, which is measured at 420 nm using a spectrophotometer:

As^3+^ + I_2_ → As^5+^ + 2 I^−^ 2 Ce^4+^ + 2 I^−^ → 2 Ce^3+^ + I_2_ (1)

A standard curve was generated by performing the SK reaction using varying concentrations of potassium iodide in distilled water as standards. Thyroid tissue lysates were normalized to 1 µg protein to quantify the intrathyroidal iodine concentrations across different genotypes. Standards and samples were prepared in triplicates and diluted in distilled water to a final volume of 62.5 µL in amber-colored Safe-Lock tubes (Eppendorf, Hamburg, Germany). Next, the tubes were incubated with 250 µL of 1 M ammonium persulfate at 100 °C for 60 min to allow oxidative digestion and release iodide from the samples. The tubes were then cooled for 30 min at room temperature, and 625 µL of arsenious acid solution (25 mM NaAsO_2_, 0.5 M H_2_SO_4_, and 0.2 M NaCl) was added and incubated at room temperature for 15 min. Subsequently, 75 µL ceric ammonium sulfate solution (15.2 mM (NH_4_)_4_Ce(SO_4_)_4_ × 2 H_2_O in 3.5 N H_2_SO_4_) was added at 30-s intervals to each tube. The tubes were incubated at room temperature for 30 min, and, thereafter, the absorbance was measured at 420 nm with an interval of 30 s between successive tubes. The average optical density from triplicate readings was used to determine the iodine concentration for respective samples using the standard curve.

### 4.7. Omics Analyses

Snap-frozen thyroid tissue was used for transcriptome (four animals per genotype at 5–8 months of age) and proteome (five animals per genotype at 5–8 months of age) analyses as described previously [86]. Briefly, the extraction of total RNA and protein was performed by modified phenol extraction with TRIzol reagent and homogenization in a bead mill dismembrator (Sartorius AG, Göttingen, Germany). After purification of total RNA, quality control was performed using an Agilent 2100 Bioanalyzer (Agilent Technologies, Basel, Switzerland). Transcriptome analyses of individual RNA samples was done using GeneChip™ Mouse Gene 1.0 ST Arrays and GeneChip^®^ Whole Transcript PLUS Reagent Kit (Affymetrix via Thermo Fisher Scientific) according to the manufacturer’s instructions. Quality of hybridizations was assessed using Expression Console software (Affymetrix via Thermo Fisher Scientific) and microarray data were analyzed using the Rosetta Resolver software system (Rosetta Biosoftware, Seattle, WA, USA).

Protein extracts were dissolved in 8 M urea/2 M thiourea solution. Protein concentrations were determined using Bradford assay (Bio-Rad Laboratories, Munich, Germany). Four µg per protein samples were reduced by 2.5 mM dithiothreitol and alkylated with 10 mM iodoacetamide. Proteolytic digestion was performed using trypsin (trypsin to protein ratio 1:25, Promega, Madison, WI, USA) at 37 °C for 17 h. The tryptic digestion was terminated by adding acetic acid to a final concentration of 1%. Subsequently, peptide mixtures were desalted using µC18 ZipTip columns (2 µg capacity, Millipore Corporation, Billerica, MA, USA) before purified peptides were analyzed using nano liquid chromatography tandem mass spectrometry (LC-MS/MS).

LC-MS/MS analyses were performed on an Ultimate 3000 nano HPLC (Thermo Fisher Scientific, Idstein, Germany) coupled to a Q Exactive^TM^ mass spectrometer (Thermo Fisher Scientific). After injection, the peptide mixture was loaded on a Thermo Scientific^TM^ Acclaim^TM^ PepMap^TM^ 100 trap column (75 μm × 20 mm, 3 μm C18 particles) and separated on an Thermo Scientific^TM^ Accucore^TM^ column (150-C18, 25 cm × 75 μm, 2.6 μm C18 particles) with a flow rate of 300 nL/min at a constant temperature of 40 °C. Reverse phase chromatography was carried out with a binary buffer system consisting of 0.1% acetic acid in water and 0.1% acetic acid in acetonitrile using a linear gradient from 5 to 25% acetonitrile. Eluting peptides were ionized with a nano electrospray ion source and analyzed in data dependent acquisition (DDA) mode. Full MS scans were recorded within a scan range of 350 to 1650 m/z at a resolution of 70,000. MS/MS data were acquired with a resolution of 17,500 for the top 10 ions with highest intensity, which were fragmented by higher energy collisional dissociation (HCD) with the collision energy set to 27.5%. Detailed information is provided in Supplemental Table S1.

LC-MS/MS raw data was processed using MaxQuant software (version 1.6.0.16). Proteins were identified by a database search in a murine UniProt/SwissProt database (rel. 2016/10) using the implemented Andromeda algorithm. Search parameters were set to a peptide mass tolerance of 10 ppm, a fragment mass tolerance of 0.02 Da, a maximum of 2 missed cleavages, carbamidomethylation of cysteine as fixed modification and methionine oxidation as variable modification. Identifications were matched between runs and peptides were validated based on a false discovery rate of 1%. For relative protein quantification, normalized label-free quantification (LFQ) intensities were imported in GeneData Analyst software (version 10.0.3) and log_10_ transformed. Gene-specific transcript and protein levels, in samples of the triple-deficient murine model, showing a fold change of ≥|1.5| over WT controls were considered for statistical analysis.

### 4.8. Statistical Analysis

Data from densitometry and fluorescence intensity measurements are presented as means ± standard deviations. For all quantifications, fold changes over WT control were determined to understand the influence of genetic alterations. Levels of significance were determined by performing one-way ANOVA with Dunnett’s correction for multiple comparisons using GraphPad Prism^TM^ (version 5.01, GraphPad Software Inc., San Diego, CA, USA). *p*-values below 0.05 were considered statistically significant. 

Data from transcriptome and proteome analyses were statistically analyzed by performing one-way ANOVA and Welch’s *t*-test, respectively, where *p*-values were corrected for multiple testing using the Benjamini Hochberg adjustment. *q*-values below 0.05 in combination with fold changes ≥ |1.5| were considered statistically significant. 

## Figures and Tables

**Table 1:** List of primary antibodies used in this study.

Antigen	Biological Source	Species Reactivity	Company/ Provider	Catalog No.	Dilution in IIF ^1^	Dilution in IB ^1^
Cathepsin B	goat	mouse	Neuromics	GT15057	1:100	1:1000
Cathepsin D	rabbit	human	Calibochem	IM-16	1:10	1:80
Cathepsin L	goat	mouse	Neuromics	GT15049	1:100	1:1000
Cystatin C	rabbit	mouse	Dr. Magnus Abrahamson		1:50	-
Cystatin D	rabbit	mouse	Dr. Magnus Abrahamson		1:50	-
Lamp1	rabbit	mouse, human, rat	Sigma Aldrich	L1418	1:100	-
LC3	rabbit	mouse, human, rat	Novus Biologicals	NB100-2331	1:100	1:500
NIS	mouse	human, rat	Abcam	ab17795	-	1:500
p62	mouse	human	Abcam	ab56146	-	1:500
Thyroglobulin	rabbit	bovine	[80]		1:30	1:5000
T3	mouse	mouse, human, rat	ICN Biochemicals	63030	1:50	-

^1^ IIF—Indirect immunofluorescence; IB—Immunoblotting.

## Data Availability

Transcriptome data presented in this study is available in GEO at https://www.ncbi.nlm.nih.gov/geo/query/acc.cgi?acc=GSE163168, reference number GSE1631168. Proteome data presented in this study is available in MassIVE at https://massive.ucsd.edu/ProteoSAFe/dataset.jsp?accession=MSV000086595, reference number MSV000086595.

## Supplementary Materials

Supplementary Materials can be found at https://www.mdpi.com/1422-0067/22/1/462/s1.

## Abbreviations

AFU	Angio-follicular units
AMPK	AMP-activated protein kinase
BSA	bovine serum albumin
CLEAR	Coordinated Lysosomal Expression and Regulation
CMF-PBS	calcium- and magnesium-free PBS
DDA	data dependent acquisition
DUOX	dual oxidase
DUOXA	dual oxidase activation maturation factor
ER	Endoplasmic reticulum
HCD	higher energy collisional dissociation
HPT	hypothalamus pituitary thyroid
LC3	microtubule-associated protein 1A/1B-light chain 3
LFQ	label-free quantification
mTORC1	mammalian target of rapamycin complex 1
NIS	sodium-iodide symporter
PBS-T	PBS containing 0.3% Tween-20
ROS	reactive oxygen species
SDS	sodium dodecyl sulfate
SK	Sandell-Kolthoff
T3	3′,3,5 triiodothyronine
T4	3,3′,5,5′ tetraiodo-L-thyronine
TFEB	transcription factor EB
Tg	thyroglobulin
TH	thyroid hormones
TPO	thyroid peroxidase
TRH	thyrotropin-releasing hormone
TSH	thyroid stimulating hormone
ULK1	Unc-51 related kinase
WT	wild-type
