# Supplementary material for: The Thyroid Hormone Transporter Mct8 Restricts Cathepsin-Mediated Thyroglobulin Processing in Male Mice through Thyroid Auto-Regulatory Mechanisms That Encompass Autophagy
Source: Int J Mol Sci. 2021 Jan 5;22(1):462. doi: 10.3390/ijms22010462 (PMC7796480; doi:10.3390/ijms22010462)
Supplement: Supplementary file 1 [file ijms-22-00462-s001.zip › Supplemental_Table_1.docx]

**Supplemental Table 1:**

| **reversed phase liquid chromatography (RPLC)** | |
| --- | --- |
| instrument | Ultimate 3000 RSLC (Thermo Fisher Scientific) |
| trap column | 75 μm inner diameter, packed with 3 μm C18 particles (Acclaim PepMap100, Thermo Scientific) |
| analytical column | Accucore 150-C18, (Thermo Fisher Scientific)  25 cm x 75 μm, 2,6 μm C18 particles, 150 Å pore size |
| buffer system | binary buffer system consisting of 0.1% acetic acid in water (buffer A) and 100% ACN in 0.1% acetic acid (buffer B) |
| flow rate | 300 nl/min |
| gradient | linear gradient of buffer B from 5% up to 25% |
| gradient duration | 120 min |
| column oven temperature | 40°C |
| **mass spectrometry (MS)** | |
| instrument | Q Exactive/ plus mass spectrometer (Thermo Fisher Scientific) |
| operation mode | data-dependent |
| **Full MS** |  |
| MS scan resolution | 70,000 |
| AGC target | 3e6 |
| maximum ion injection time for the MS scan | 120 ms |
| Scan range | 300 to 1650 m/z |
| Spectra data type | profile |
| **dd-MS2** |  |
| Resolution | 17,500 |
| MS/MS AGC target | 2e5 |
| maximum ion injection time for the MS/MS scans | 120 ms |
| Spectra data type | centroid |
| selection for MS/MS | 10 most abundant isotope patterns with charge ≥2 from the survey scan |
| isolation window | 3 m/z |
| Fixed first mass | 100 m/z |
| dissociation mode | higher energy collisional dissociation (HCD) |
| normalized collision energy | 27.5% |
| dynamic exclusion | 30 s |
| Charge exclusion | 1,>6 |
