# Supplementary figures and images for: The Thyroid Hormone Transporter Mct8 Restricts Cathepsin-Mediated Thyroglobulin Processing in Male Mice through Thyroid Auto-Regulatory Mechanisms That Encompass Autophagy
Source: Int J Mol Sci. 2021 Jan 5;22(1):462. doi: 10.3390/ijms22010462 (PMC7796480; doi:10.3390/ijms22010462)

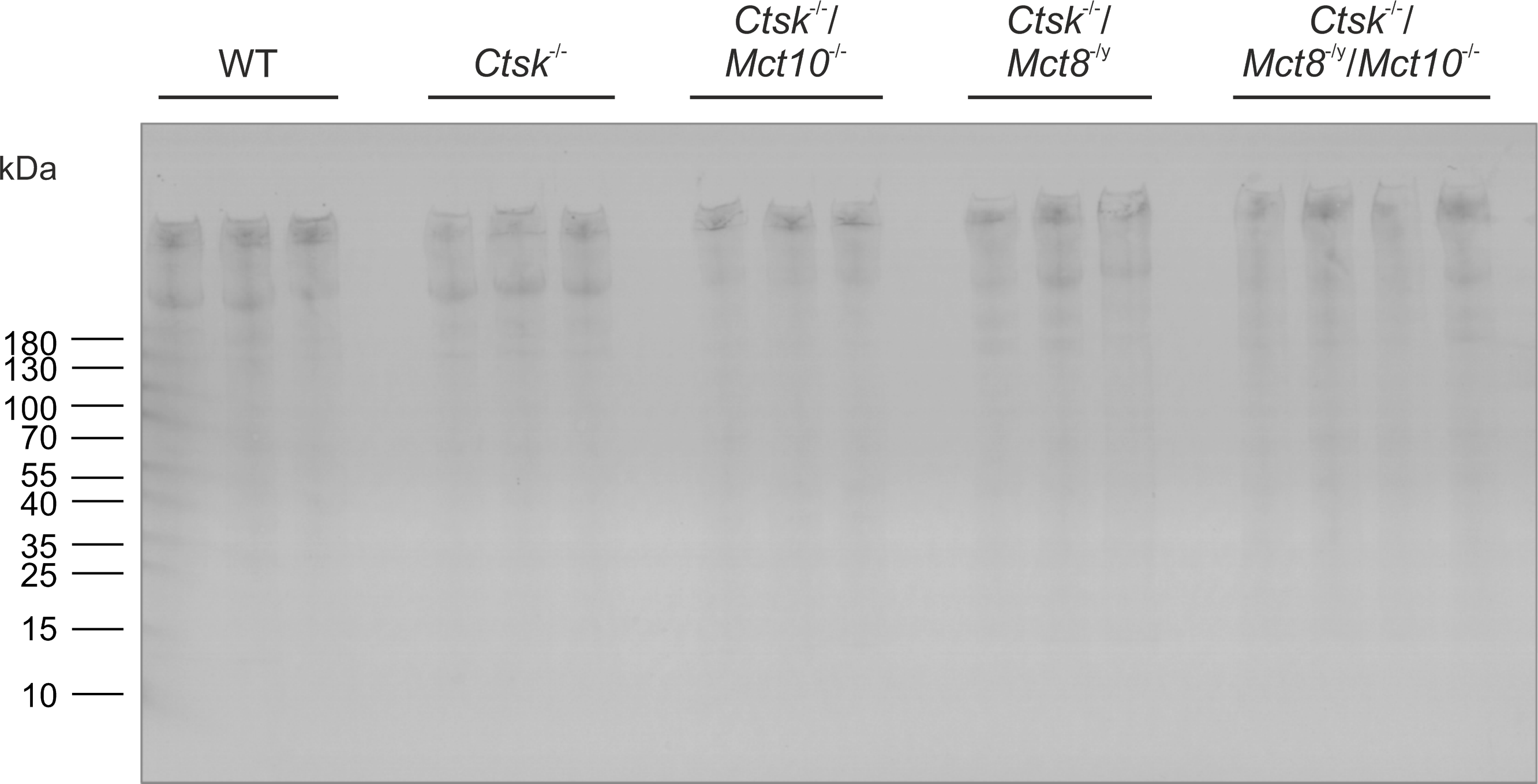

Supplement: Supplementary file 1 [file ijms-22-00462-s001.zip › Figure S1-Ponceau for Figure 2A.tif]

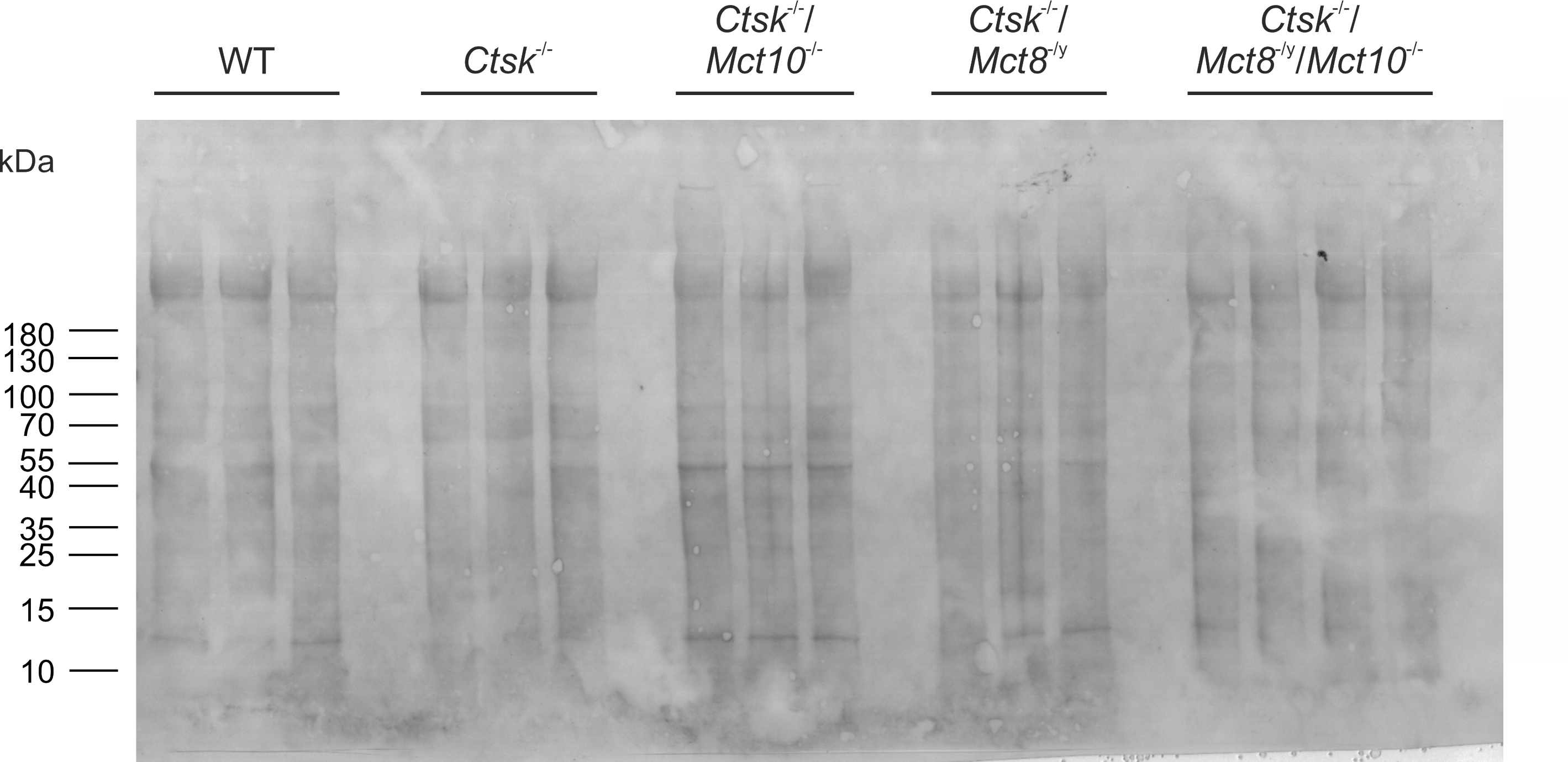

Supplement: Supplementary file 1 [file ijms-22-00462-s001.zip › Figure S2-Ponceau for Figures 4A and 10C.tif]

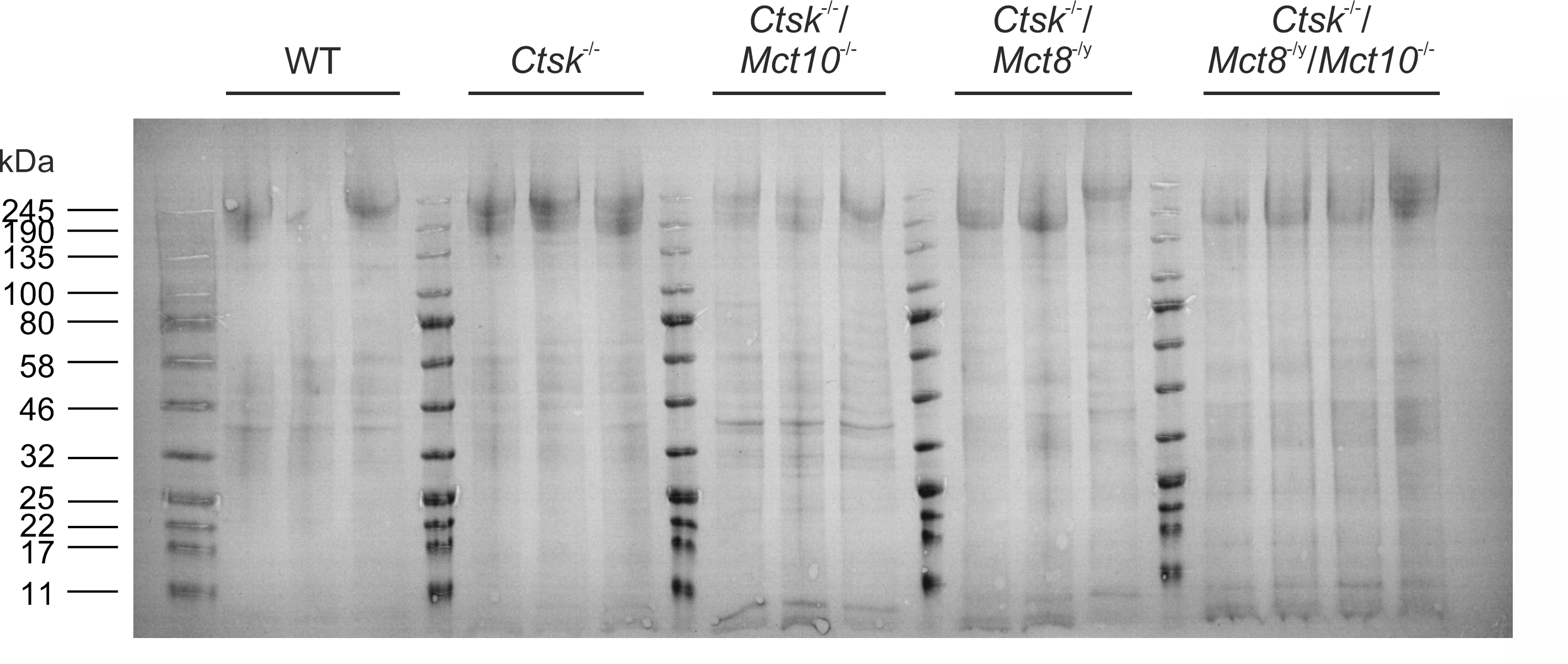

Supplement: Supplementary file 1 [file ijms-22-00462-s001.zip › Figure S3-Ponceau for Figures 5A and Figures 7A-C.tif]

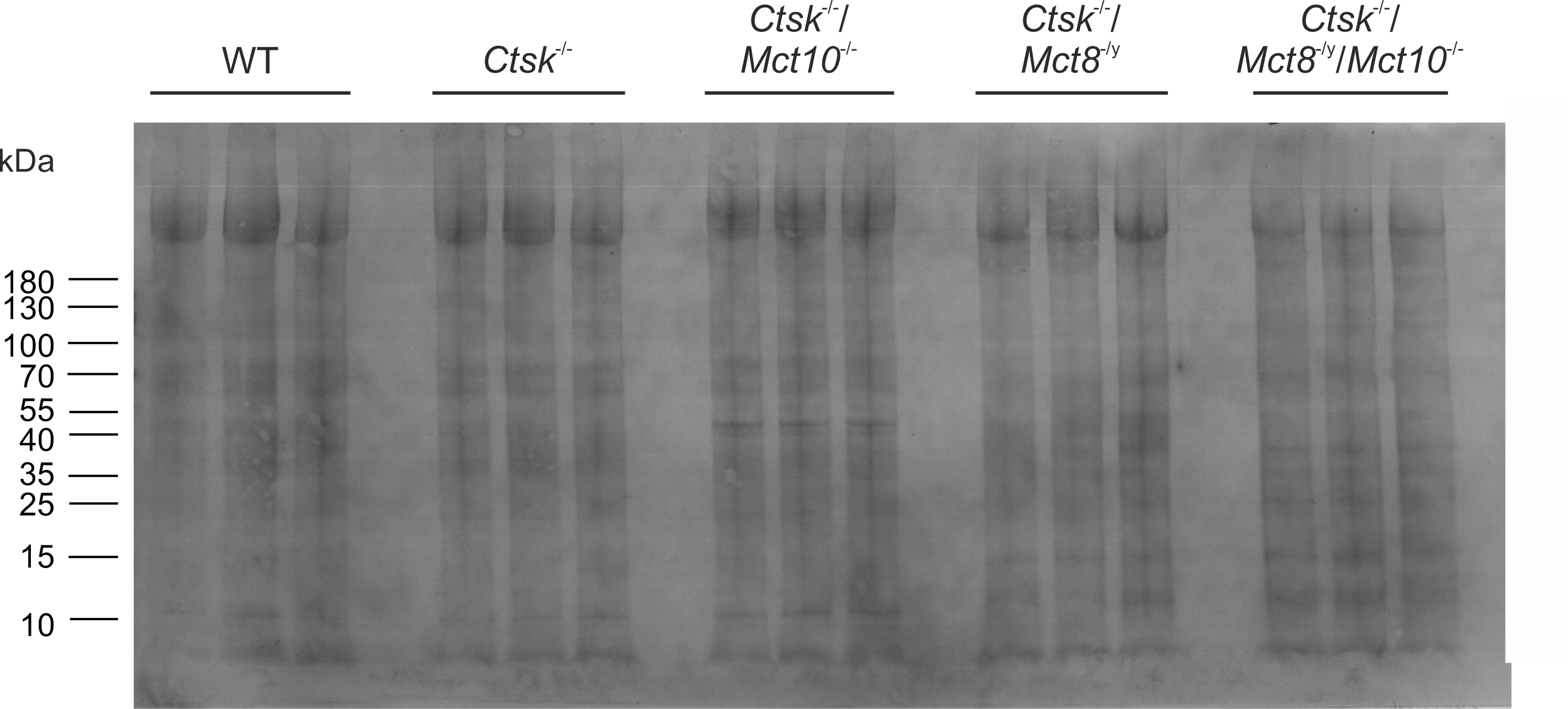

Supplement: Supplementary file 1 [file ijms-22-00462-s001.zip › Figure S4-Ponceau Figure 10B.tif]

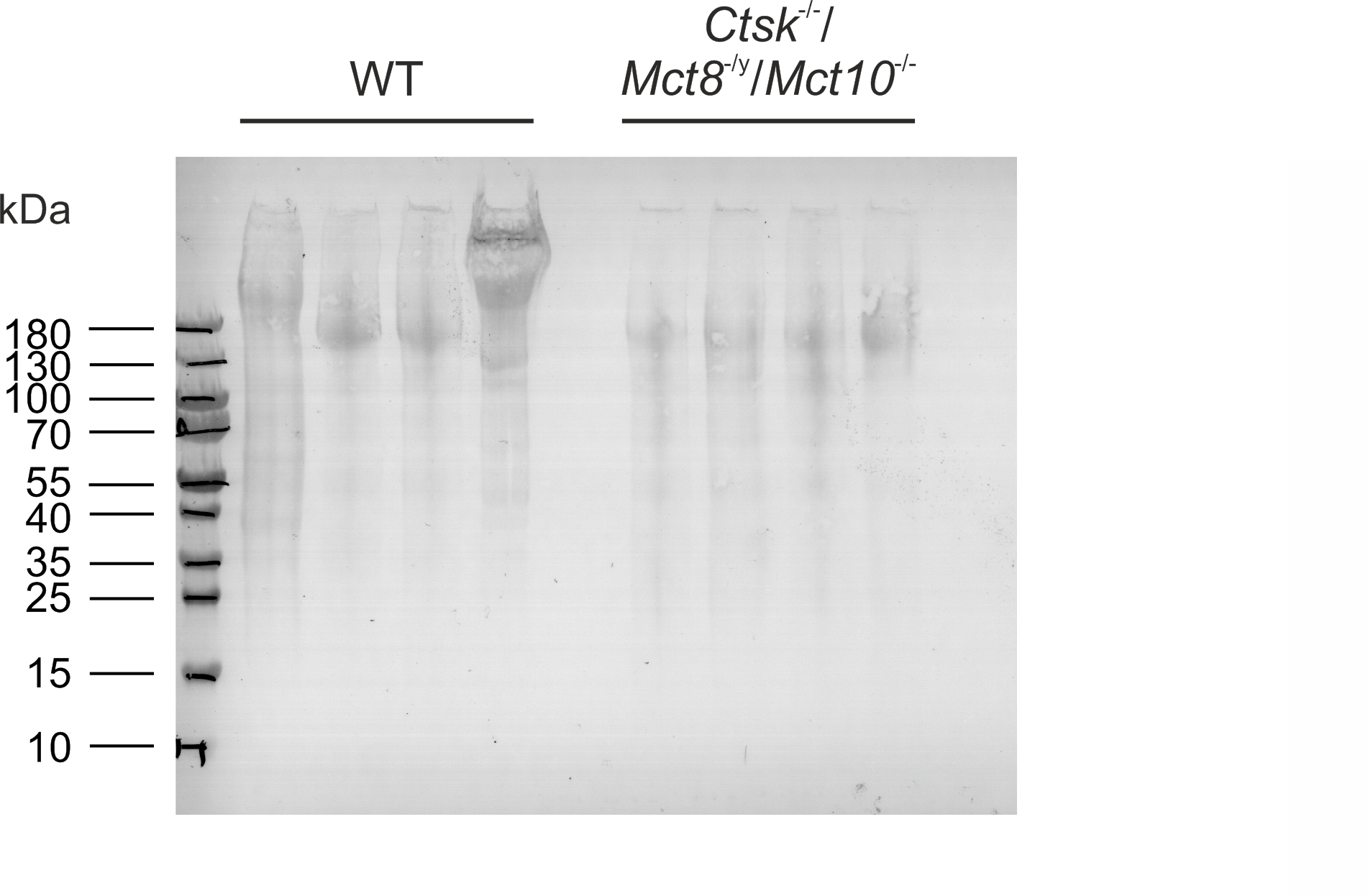

Supplement: Supplementary file 1 [file ijms-22-00462-s001.zip › Figure S5_Ponceau for Figure 11C.tif]
